# Cost-Effectiveness of Large-Bore Mechanical Thrombectomy Versus Catheter-Directed Thrombolysis for Intermediate-Risk Pulmonary Embolism: A PEERLESS Randomized Controlled Trial Post Hoc Analysis

**DOI:** 10.1016/j.jscai.2026.105578

**Published:** 2026-08-25

**Authors:** Samuel Horr, Orestis Pappas, Ripal T. Gandhi, Keith Pereira, Jay Giri, Sameer J. Khandhar, Ezana M. Azene, Joseph Campbell, Jonathan Belsey, Rick Wat, Jarrod Collins, Wissam A. Jaber, Carin F. Gonsalves, Stefan Stortecky

**Affiliations:** a Department of Cardiovascular Medicine, TriStar Centennial Medical Center, Nashville, Tennessee; b Allegheny Health Network, Erie, Pennsylvania; c Miami Cardiac and Vascular Institute, Baptist Health South Florida, Miami, Florida; d Department of Radiology, Saint Louis University, Saint Louis, Missouri; e Cardiovascular Medicine Division, Perelman School of Medicine, University of Pennsylvania, Philadelphia, Pennsylvania; f Emplify Health, La Crosse, Wisconsin; g Department of Cardiology, OhioHealth Riverside Methodist Hospital, Columbus, Ohio; h JB Medical Limited, London, United Kingdom; i Inari Medical Division of Stryker Corporation, Irvine, California; j Department of Medicine, Emory University Hospital, Atlanta, Georgia; k Division of Interventional Radiology, Thomas Jefferson University Hospitals, Philadelphia, Pennsylvania; l Department of Cardiology, Inselspital, Bern University Hospital, University of Bern, Bern, Switzerland; m Department of Cardiology, Bürgerspital Solothurn, Solothurner Spitäler AG (soH), Solothurn, Switzerland

**Keywords:** catheter-directed thrombolysis, cost-effectiveness, large-bore mechanical thrombectomy, pulmonary embolism, venous thromboembolism

## Abstract

**Background:**

This study evaluates the cost-effectiveness of large-bore mechanical thrombectomy (LBMT) compared with catheter-directed thrombolysis (CDT) for the treatment of intermediate-risk pulmonary embolism (PE).

**Methods:**

A combined decision tree and Markov model was developed to estimate lifetime health care costs, quality-adjusted life years (QALY), and net monetary benefit from the perspective of the United States health care system. Event probabilities during PE management were derived from the PEERLESS randomized controlled trial. Resource utilization, costs, and long-term event probabilities were established from published literature. Scenario analyses and probabilistic sensitivity analyses were performed to characterize uncertainty.

**Results:**

In the base case analysis, LBMT was cost-saving to CDT (ie, lower costs and higher QALY), with a net monetary benefit of $5850 for the willingness-to-pay threshold of $120,000/QALY for LBMT when compared to CDT. In the PE management phase, LBMT yielded a net $4755 cost-saving and 0.009 QALY gained per patient vs CDT, driven in large part by reduced intensive care unit utilization (–$6454), shorter hospital stay (–$2217), avoided thrombolytic costs (–$4068), and fewer 30-day readmissions (–$1134). The long-term care phase yielded a total of $32,012 additional costs per patient over the lifetime with no incremental difference in costs or QALY between groups. These additional costs were driven by venous thromboembolism–related death ($12,377), chronic thromboembolic pulmonary hypertension diagnosis and treatment ($8080), postacute anticoagulation management ($4512), and recurrent venous thromboembolism ($3289). LBMT remained cost-saving across scenarios evaluating CDT cost assumptions and device-specific chronic thromboembolic pulmonary hypertension rates. Probabilistic sensitivity analyses confirmed the robustness of these findings across multiple parameter ranges.

**Conclusions:**

Intervention with LBMT in intermediate-risk PE patients in the United States is economically attractive when compared with CDT, resulting in reduced costs and marginally improved patient benefit.

## Introduction

Acute pulmonary embolism (PE) is a common condition associated with significant morbidity and mortality. Advanced reperfusion techniques, including catheter-directed thrombolysis (CDT) and large-bore mechanical thrombectomy (LBMT), are increasingly implemented to reduce thrombus burden in patients who deteriorate on anticoagulation (AC) or when multidisciplinary care teams determine that interventional treatment is in the patient’s best interest.^1^^,^^2^ CDT and LBMT procedures are employed in conjunction with AC therapy to either dissolve thrombus through local infusion of a thrombolytic agent or mechanically extract thrombus from the pulmonary vasculature, respectively. Clinical implementation of these interventional approaches for treating intermediate-risk PE has accelerated in recent years as their strong safety and effectiveness profiles have been characterized in clinical studies.^3^, ^4^, ^5^, ^6^, ^7^, ^8^, ^9^

Commonly, in health economic evaluations, a health care strategy is considered economically attractive when the incremental cost of the treatment per unit of benefit gained by the patient is below an established willingness-to-pay (WTP) threshold.^10^ Despite the increasing clinical utilization of CDT and LBMT, direct assessment of their relative cost-effectiveness is lacking. Prior studies have evaluated the comparative costs of these treatment strategies, often using retrospective or literature-based designs and variable cost-accounting methods, which yielded mixed findings and lacked assessment of cost-effectiveness.^11^, ^12^, ^13^ Consequently, based on currently available literature, it remains unclear whether the cost-benefit differences between CDT and LBMT demonstrate relative value and economically justify the preference of a particular interventional strategy.

The PEERLESS randomized controlled trial (RCT) recently compared clinical outcomes through 30 days for intermediate-risk PE patients treated with either LBMT with the FlowTriever System (Inari Medical) or CDT per local standards.^3^ Trial results indicated that patients randomized to LBMT had significantly reduced rates of clinical deterioration and/or escalation to bail out therapy (CDBO), postprocedure intensive care unit (ICU) utilization, and all-cause readmission, along with earlier recovery as measured by patient-reported symptoms and imaging findings. However, there were no significant differences in all-cause mortality, intracranial hemorrhage (ICH), or major bleeding outcomes through hospital discharge. Building on these findings, we apply clinical outcomes from the PEERLESS RCT as the foundation for a cost-effectiveness analysis. This study examines the economics of LBMT vs CDT for treating intermediate-risk PE through a lifetime horizon in the United States.

## Materials and methods

This analysis evaluated the cost-effectiveness of LBMT with the FlowTriever system and CDT with local standard of care in a hypothetical cohort of 300,000 intermediate-risk PE patients from the perspective of the US health care system over a lifetime horizon. Model parameters were established from the PEERLESS RCT and published literature, as referenced below. The analysis was constructed in Excel v2503 (Microsoft Corporation). The study conforms to the quality and reporting standards as described in the Criteria for Health Economic Quality Evaluation tool and the Consolidated Health Economic Evaluation Reporting Standards (CHEERS) statement (Supplemental Tables S1-3).^14^^,^^15^ An impact inventory detailing which value criteria were included was developed in accordance with the 2016 guidelines from the Second Panel on Cost-Effectiveness in Health and Medicine (Supplemental Tables S4).^16^

### Model overview

A combined modeling approach was utilized, employing a decision tree to simulate the initial PE management and immediate follow-up period (ie, PE management phase) before transitioning to a Markov state model of long-term health status (ie, long-term care phase) (Figure 1). This structure allowed for an estimation of individual patient status and derived costs as hypothetical patients progressed through the model. The model’s structure was informed by established cost-utility models from the United States and the United Kingdom, ensuring methodological consistency with validated frameworks.^17^, ^18^, ^19^, ^20^

**Figure 1:**
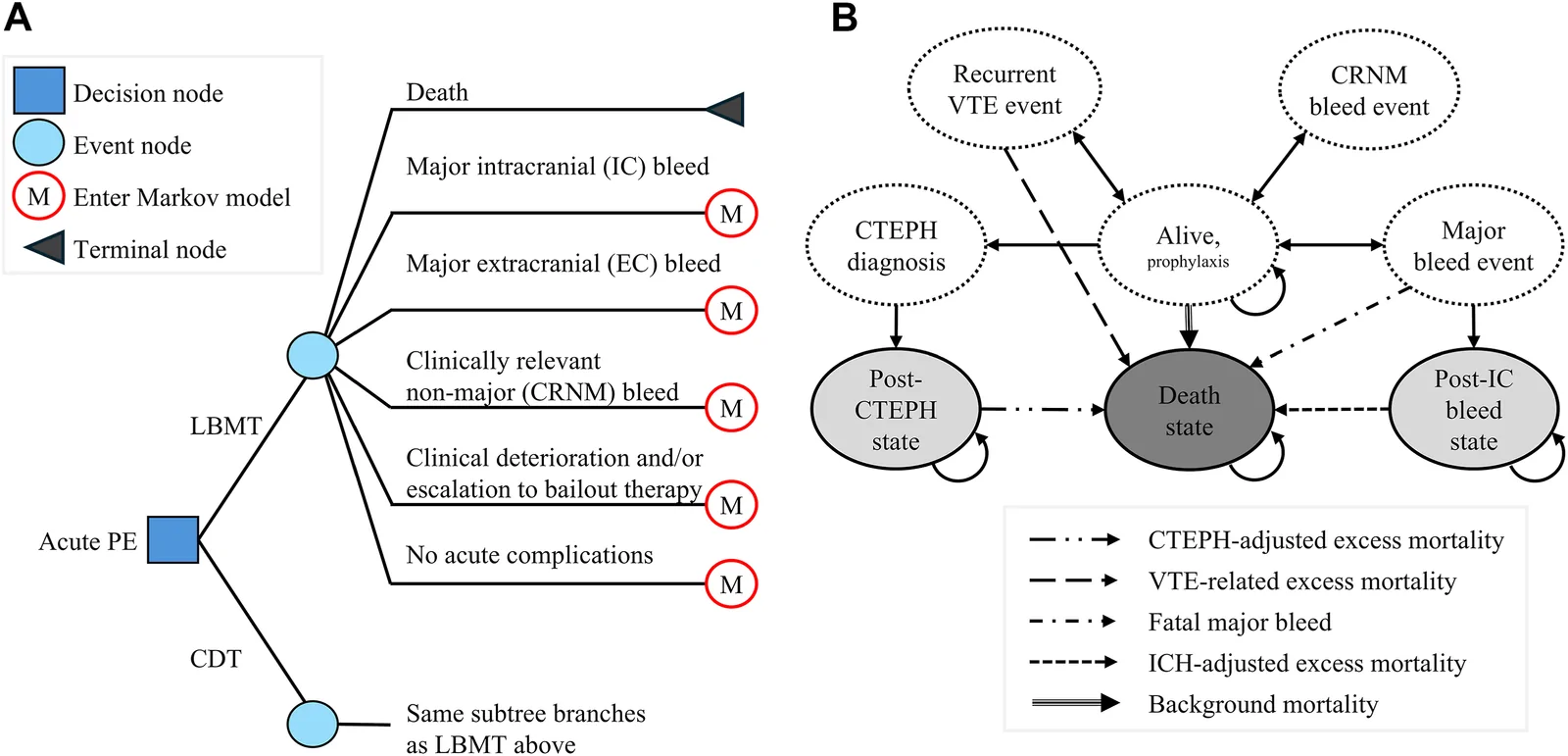
**Model structure.** Combined decision tree-Markov modeling diagram. (A) The decision tree illustrates the probabilities of no acute complications, short-term complications resulting from pulmonary embolism (PE) management, and death. (B) The Markov model incorporates possible health states through a lifetime horizon for those patients surviving the PE management phase. Arrows indicate possible health state transitions. Health states displayed in white-dashed, light gray, and dark gray represent transient, semiabsorbing, and absorbing health states, respectively. CDT, catheter-directed thrombolysis; CRNM, clinically relevant nonmajor; CTEPH, chronic thromboembolic pulmonary hypertension; EC, extracranial; IC, intracranial; ICH, intracranial hemorrhage; LBMT, large-bore mechanical thrombectomy; VTE, venous thromboembolism.

### PE management phase

Complicating health states that may arise during the PE management phase were modeled using a decision tree (Figure 1A). After being assigned to a treatment group (ie, LBMT or CDT), hypothetical patients in the model could experience one of several potential acute clinical outcomes, listed in order of decreasing severity: death, major intracranial (IC) bleeding, major extracranial bleeding, CDBO, clinically relevant nonmajor bleeding, or no acute complications, consistent with previously published economic evaluations.^21^, ^22^, ^23^ Decision tree event nodes were treated as mutually exclusive, meaning that event probabilities were parameterized based on PEERLESS patients’ highest severity events and remaining patients were captured as having no acute complications. Each node represents a possible patient outcome from which cost and benefit were estimated, encompassing the PE procedure, acute clinical outcomes, hospital and ICU stay, 30-day readmission, and 30-day mortality. Both treatment groups were assumed to receive AC with direct oral AC for at least 3 months postprocedure, consistent with US and European clinical guidelines.^1^^,^^24^

Clinical outcome rates in the PE management phase were established from the PEERLESS RCT.^3^^,^^25^ The trial design and methods of the PEERLESS RCT (ClinicalTrials.gov; NCT05111613) have been previously reported in detail.^3^^,^^25^ The study enrolled 550 randomized patients from 57 centers across the United States and Europe between February 2022 and February 2024.^3^ Eligible patients were ≥18 years of age, had intermediate-risk PE as defined by the European Society of Cardiology guidelines, and additional clinical risk factors.^1^ Patients were randomized 1:1 to receive either LBMT using the FlowTriever system or CDT using local standard of care. As such, differing CDT devices were permitted within the study protocol (detailed in Supplemental Table S5).^3^ Patient outcomes were collected at 24 hours (±8 hours), hospital discharge, and 30 days (+15 days). The primary end point of the trial was a hierarchal composite win ratio of the following outcomes evaluated at the earlier of discharge or 7 days postprocedure: (1) all-cause mortality, (2) ICH, (3) major bleeding as defined by the International Society for Thrombosis and Haemostasis,^26^ (4) CDBO, and (5) postprocedural ICU admission and length of stay (LOS). The complete end point definitions and additional secondary end points have been previously reported.^3^^,^^25^ The study obtained institutional review board or ethics committee approval at each site, and all patients provided informed consent.

In the PEERLESS RCT, baseline demographic characteristics were comparable between the LBMT and CDT cohorts (mean age 63.7 vs 61.2 years and 54.4% vs 51.4% male, respectively).^3^ The study population was predominantly intermediate-high-risk according to the European Society of Cardiology guidelines, with 94.7% of patients demonstrating both RV dysfunction on imaging and elevated cardiac troponins.^1^ Additionally, the study population had a low bleeding risk, with 4.2% presenting with relative contraindications to thrombolytics. Procedural timing was similar between groups, with a mean time from hospital presentation to catheter insertion of 22.3 ± 17.7 hours for LBMT patients and 24.9 ± 19.7 hours for CDT patients.

### Long-term care phase

Disease progression beyond the PE management phase was modeled using a Markov state model (Figure 1B) over a lifetime horizon. The model applied a 3-month cycle length, with health state transitions occurring at the midpoint of each cycle. After accounting for 30-day background mortality from the PE management phase, surviving patients from the decision tree entered the Markov model and could remain in their current health state or transition between health states at each model cycle. The alive health state, chronic thromboembolic pulmonary hypertension (CTEPH) diagnosis, recurrent venous thromboembolism (VTE) event, clinically relevant nonmajor bleed event, and major bleed event were modeled as transient health states. The post-IC bleed and post-CTEPH health states were treated as semiabsorbing states and represent conditions that patients enter following an IC bleed or developing CTEPH, respectively, and remain until transitioning to the death state. The alive health state, recurrent VTE event, major bleed event, post-IC bleed health state, and post-CTEPH health state could transition to the death state, which serves as the final absorbing health state and accumulated patients until the lifetime horizon was completed for all hypothetical patients.

Clinical outcomes in the long-term care phase were based on published literature.^17^^,^^18^^,^^27^, ^28^, ^29^, ^30^, ^31^, ^32^ Long-term health states were applied uniformly to both treatment groups, assuming that all patients received standard treatment with direct oral AC, with event rates derived from the AMPLIFY RCT, the AMPLIFY-EXT RCT, and a systematic review and meta-analysis of CTEPH incidence following acute PE.^17^^,^^18^^,^^27^^,^^29^^,^^30^^,^^32^ The model assumes that all incident CTEPH cases occur within the first 5 years posttreatment.^33^ The time-dependent risk of recurrent VTE and VTE-related death beyond 18 months was informed from a prospective 10-year follow-up cohort study that tracked VTE outcomes following cessation of AC.^31^ Background mortality was modeled using age- and sex-specific US life tables, with hazard ratios from retrospective studies applied to adjust for the increased mortality risk associated with recurrent VTE, CTEPH, and IC bleed health states.^18^^,^^34^

Health state utilities and disutilities were conservatively derived from published literature to estimate quality of life impact. The complete list of model parameters, including probabilities of acute complications and long-term health state transitions as well as health state utilities and disutilities, is detailed in Supplemental Tables S6-9, respectively. For clarity and to highlight the clinical events incorporated into the model, Table 1^3^ summarizes the parameterization of key complication rates in each model phase.

**Table 1:** Parameterization of key model health states from clinical outcomes

	LBMT	CDT	Reference
PE management phase			
All-cause mortality^a^	0.0%	0.4%	PEERLESS^3^,^b^
Major IC bleed^a^	0.7%	0.4%	PEERLESS^3^,^b^
Major EC bleed^a^	6.2%	6.2%	PEERLESS^3^,^b^
Clinical deterioration and/or escalation to bailout^a^	1.5%	4.3%	PEERLESS^3^,^b^
CRNM bleed^a^	2.6%	2.5%	PEERLESS^3^,^b^
No complications	89.1%	86.2%	b
Long-term care phase (per 3-mo model cycle increment, range shown for 0-5 y)			
Major IC bleed^c^^,^^d^	0.01%-0.05%		Supplemental Table S8
Major EC bleed^c^^,^^d^	0.09%-0.31%		Supplemental Table S8
CRNM bleed^c^^,^^d^	0.57%-2.65%		Supplemental Table S8
Fatal major bleed^c^^,^^d^	0.02%-0.06%		Supplemental Table S8
Recurrent VTE^c^^,^^d^	0.42%-2.44%		Supplemental Table S8
VTE-related mortality^c^^,^^d^	0.10%-2.00%		Supplemental Table S8
CTEPH^c^^,^^d^	0.12%		Supplemental Table S8

Values are model parameters.

CDT, catheter-directed thrombolysis; CRNM, clinically relevant nonmajor; CTEPH, chronic thromboembolic pulmonary hypertension; EC, extracranial; IC, intracranial; LBMT, large-bore mechanical thrombectomy; PE, pulmonary embolism; VTE, venous thromboembolism.

a Measured at the sooner of hospital discharge or 7 days following the procedure.

b Event probabilities were assumed based on each patient’s highest severity event in order of all-cause mortality, major IC bleed, major EC bleed, clinical deterioration and/or escalation to bail out therapy, and CRNM bleed, with the remainder captured as no complications.

c Approximations of model cycle rates that may vary based on time from the PE treatment.

d Rates applied equally to both groups assuming continued anticoagulation management following PE treatment.

### Cost parameters

Health care costs for the PE management phase were sourced from a combination of resource-based accounts, hospital billing data, and event-based methods (eg, applying average costs for readmissions rather than trial-specific data), consistent with prior economic analyses.^13^^,^^35^ Costs and resource use were evaluated in terms of 2024 US dollars ($). Costs from years preceding 2024 were converted to 2024 US dollars using the medical care consumer price index.^36^ An annual discount rate of 3% was applied to account for time preference.^16^^,^^37^ Comprehensive details of the costing methods and estimates are available in the Supplemental Methods: Determination of medical care costs and Supplemental Tables S10-13.

### Analytic end points

The analytic end points of this study included costs, quality-adjusted life years (QALY) and life years (LY), incremental cost-effectiveness ratio (ICER), and net monetary benefit (NMB). For reference, QALY represent the gain in patient benefit following treatment. The ICER represents the cost per additional QALY gained when comparing 2 treatments. The NMB summarizes the monetary value of a given treatment by scaling the WTP threshold by the incremental benefit and subtracting the incremental cost. When comparing 2 treatments, a positive NMB indicates that the cost to gain benefit is less than the maximum amount which is willing to be paid and that the treatment is, therefore, cost effective.

### Base case analysis

The base case analysis reflects the primary assumptions used to model long-term cost-effectiveness in this study. For the initial PE management phase, clinical event rates and the distribution of CDT device use were sourced from the PEERLESS RCT (Supplemental Tables S5). For the long-term care phase, complication rates were assumed to be equal for both treatment groups, providing a treatment-agnostic projection of long-term outcomes.

### Sensitivity analyses

Univariate and probabilistic sensitivity analyses were performed to assess the impact that parameter uncertainty had on cost-effectiveness. The univariate sensitivity analysis independently varied the costs, health state probabilities, and health utilities based on the upper and lower bounds of the interquartile range, published high and low values from literature, or by varying the parameter by ±10% (except for the disutility of bailout to any treatment, which applied ±50%) when additional sources were unavailable. Further, a Monte Carlo simulation was implemented to probabilistically evaluate parameter uncertainty across 5000 simulations while simultaneously varying all parameters. The assumed upper and lower bounds and probabilistic distributions for each model parameter are available in the Supplemental Material.

### Scenario analyses and model validation

Scenario analyses were conducted to explore uncertainty in key model assumptions. First, additional univariate sensitivity analysis was performed to evaluate the impact of differential CDT device selection on cost-effectiveness. Although the base case analysis assumed rates of CDT device utilization from the PEERLESS trial, we investigated 2 additional scenarios that assume exclusive use of the lowest- and highest-cost CDT devices, standard side-hole catheter and ultrasound-facilitated CDT, respectively. Second, the base case analysis was adjusted to implement device-specific long-term complication rates to observe the potential impact of differential CTEPH development. A systematic literature review was conducted to identify studies reporting device-specific long-term risk of CTEPH following LBMT or CDT, details of which are reported in Supplemental Methods. For this scenario, device-specific CTEPH rates were informed from a real-world electronic health record study identified via the systematic review that compared 5-year outcomes of mechanical thrombectomy and CDT.^33^

Model validation was conducted following ISPOR-SMDM good practice recommendations for health economic modeling, including checks for face, internal, cross, and external validity, as described in Supplemental Methods.^38^

## Results

Results of the deterministic base case analysis are reported in Table 2.^3^ The total discounted lifetime cost and QALY per patient were $68,950 and 6.374 for LBMT and $73,705 and 6.364 for CDT, respectively. Treatment with LBMT was, therefore, less costly (–$4755) and more effective (+0.009 QALY) compared to CDT. This state of dominance for LBMT (ie, lower costs and greater benefit) implies cost-effectiveness at any WTP threshold. When a cost-effectiveness evaluation results in a state of dominance, the ICER loses meaning and is not explicitly reported.^10^ At a WTP threshold of $120,000/QALY, the NMB for the base case analysis was $5850, indicating the additional value that LBMT provides per patient compared to CDT (Central Illustration).^10^

**Table 2:** Discounted cost-effectiveness of LBMT vs CDT over lifetime horizon for the base case. analysis

	LBMT	CDT	Difference	Result
Lifetime costs, $	68,950	73,705	(4755)	–
QALY	6.374	6.364	0.009	LBMT is cost-saving
LY	7.497	7.496	0.000	–
NMB for LBMT,^a^ $	–	–	–	5850

Values are discrete model outputs.

Values in parentheses indicate a negative number.

CDT, catheter-directed thrombolysis; LBMT, large-bore mechanical thrombectomy; LY, life year; NMB, net monetary benefit; QALY, quality-adjusted life year.

a Assuming <$120,000/QALY willingness-to-pay threshold.

**Central Illustration:**
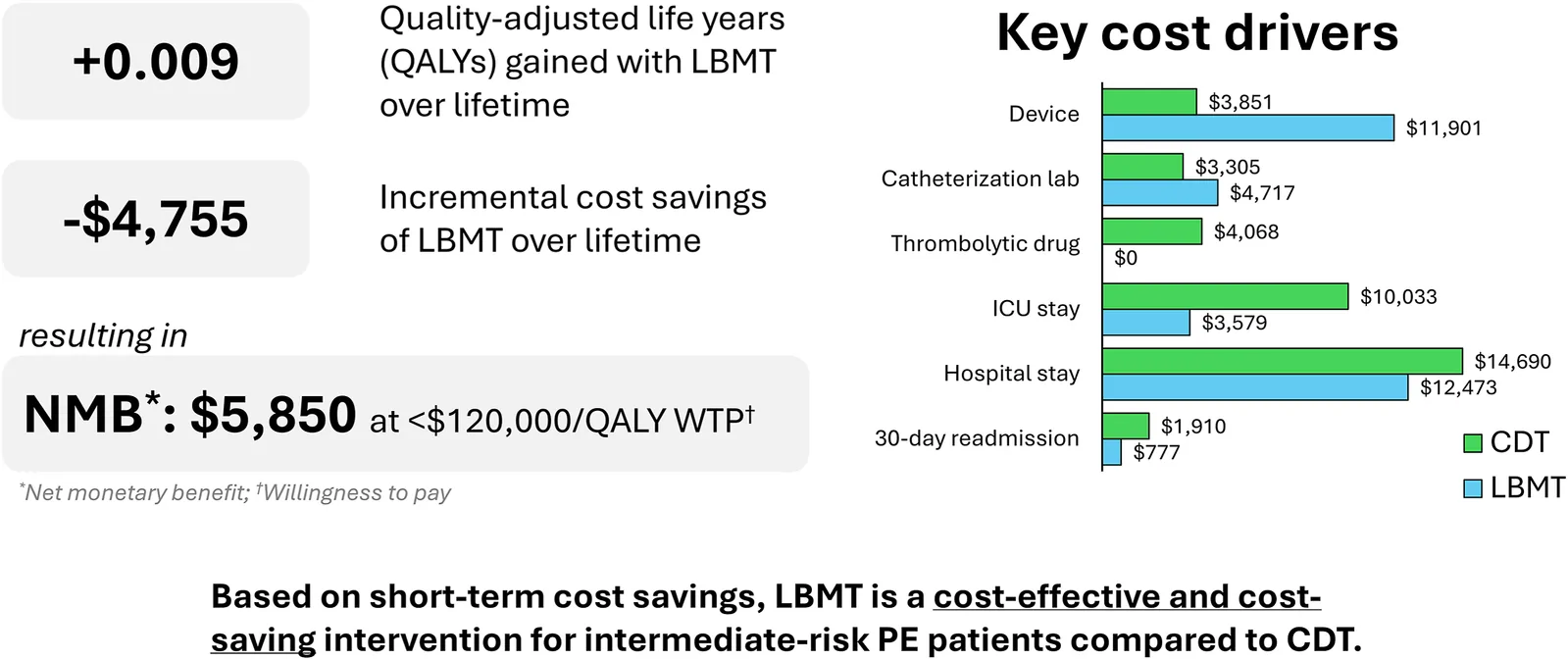
**Cost-effectiveness oflarge-boremechanical thrombectomy (LBMT) compared tocatheter-directedthrombolysis (CDT) inintermediate-riskpulmonary embolism (PE) in the United States.** Key cost drivers related to short-term resource management in the pulmonary embolism management phase. ICU, intensive care unit; NMB, net monetary benefit; QALY, quality-adjusted life years; WTP, willingness-to-pay.

### PE management phase

Although the LBMT group incurred higher upfront costs from the device (+$8050) and procedure duration (+$1412), these were offset by greater cumulative expenses in the CDT group driven by longer ICU stays (+$6454), use of thrombolytics (+$4068), longer hospital stays (+$2217), and higher rates of 30-day readmissions (+$1134) and clinical deterioration and/or escalation to bail out (+$473). A detailed breakdown of these costs is available in Table 3. These same differences in-hospital and ICU stay, as well as 30-day mortality and readmission outcomes, resulted in the incremental QALY gain of 0.009 for the LBMT group.

**Table 3:** Categorical cost subtotals for LBMT vs CDT from base case analysis

Item or event costs, $	LBMT	CDT	Difference
PE management phase			
Device	11,901	3851	8050
Catheterization lab	4717	3305	1412
Other procedural costs	437	371	66
Thrombolytic drug	0	4068	(4068)
ICU stay	3579	10,033	(6454)
Hospital stay	12,473	14,690	(2217)
Other in-hospital costs	1160	1278	(118)
Major IC bleed	337	167	170
Major EC bleed	1330	1320	10
CRNM bleed	137	136	1
Clinical deterioration and/or escalation to bail out	91	563	(473)
30-d readmission costs	777	1910	(1134)
PE management phase subtotal	36,938	41,693	(4755)
Long-term care phase			
Anticoagulation therapy	4512	4512	0
Recurrent VTE	3289	3289	0
VTE-related death	12,377	12,377	0
Fatal major bleed	173	173	0
IC bleed	1577	1577	0
EC bleed	737	737	0
CRNM bleed	1267	1267	0
CTEPH	8080	8080	0
Long-term phase subtotal	32,012	32,012	0
Lifetime			
Cumulative costs	68,950	73,705	(4755)

PE management phase costs assume device-specific clinical event rates from the PEERLESS randomized controlled trial. Long-term care phase costs assume equal clinical event rates for both treatment groups.

Values are discrete model outputs.

Values in parentheses indicate a negative number.

CDT, catheter-directed thrombolysis; CRNM, clinically relevant nonmajor; CTEPH, chronic thromboembolic pulmonary hypertension; EC, extracranial; IC, intracranial; ICU, intensive care unit; LBMT, large-bore mechanical thrombectomy; PE, pulmonary embolism; VTE, venous thromboembolism.

### Long-term care phase

In the long-term care phase of the model, because equal event rates were assumed for both treatment groups, there was no incremental difference in costs or QALY between the strategies. However, the model did estimate additional long-term care costs of $32,012 per patient following initial PE management (Table 3). These lifetime expenses were driven by VTE-related death ($12,377), CTEPH diagnosis and treatment ($8080), postacute AC management ($4512), and recurrent VTE ($3289).

### Sensitivity analyses

The univariate sensitivity analysis results are reported as the change to the NMB in a tornado diagram (Figure 2). The NMB was most sensitive to the cost of the ICU stay, cost of the LBMT device, cost of thrombolytic drugs, length of hospital stay, and proportion with 30-day readmissions. LBMT maintained a positive NMB when cost and utility parameters were evaluated at extreme high and low values.

**Figure 2:**
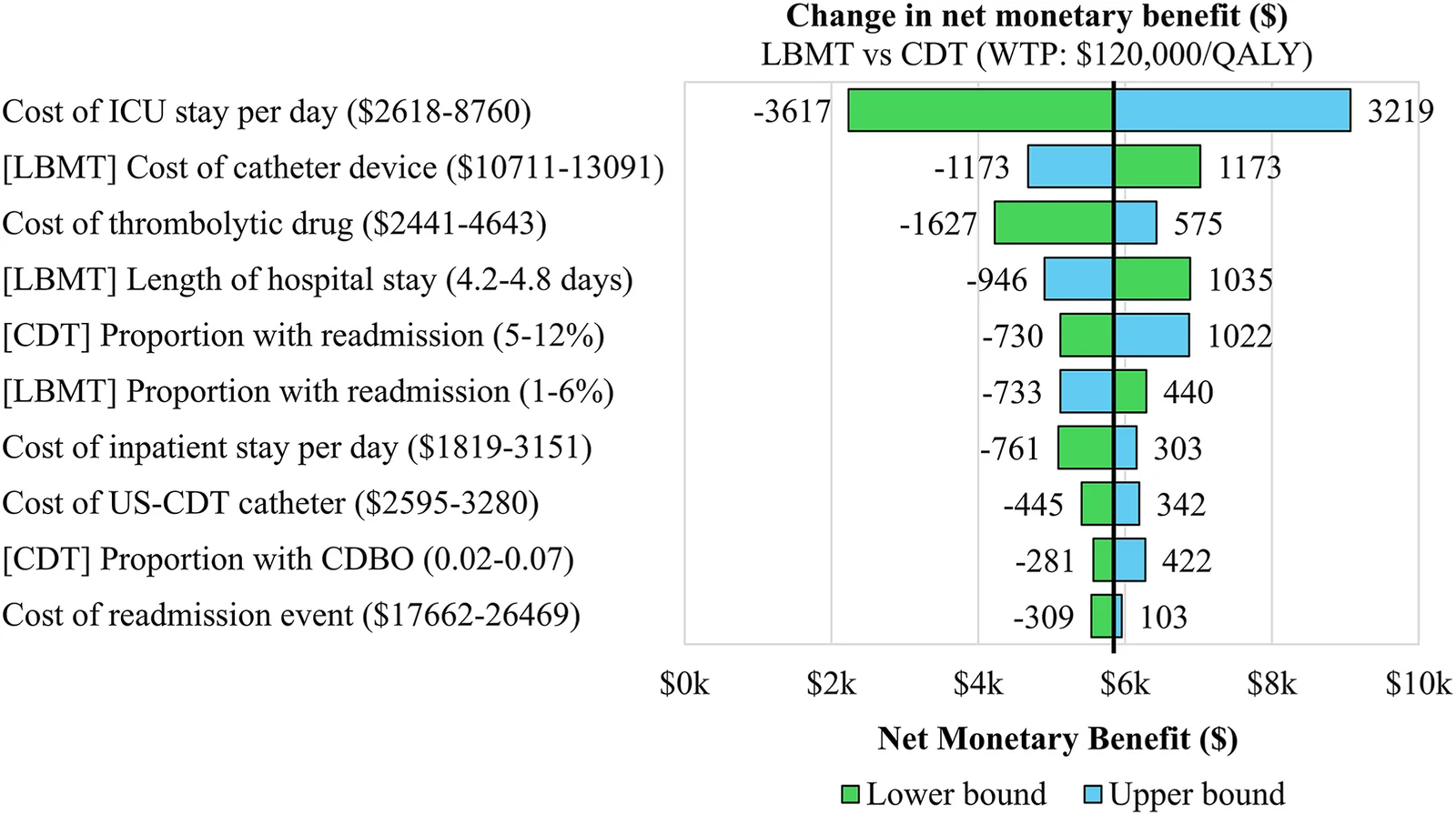
**Univariate sensitivity analysis.** Tornado diagram of univariate sensitivity analysis for large-bore mechanical thrombectomy (LBMT) vs catheter-directed thrombolysis (CDT), reporting the influence of individual model variables on net monetary benefit. The range shown in parentheses for each variable represents the evaluated upper and lower bounds, detailed in the Supplemental Materials. The vertical black line indicates the base case net monetary benefit of $5850. CDBO, clinical deterioration and/or escalation to bail out therapy; ICU, intensive care unit; QALY, quality-adjusted life year; US-CDT, ultrasound-facilitated catheter-directed thrombolysis; WTP, willingness-to-pay.

The probabilistic sensitivity analysis results are reported in Figure 3. LBMT was cost effective to CDT at the $120,000/QALY WTP threshold in 98.2% (4908/5000) of simulations, of which: LBMT was cost-saving (ie, less costly and more effective) in 85.8% (4291/5000), less costly and less effective in 10.8% (540/5000), and more costly and more effective in 1.5% (77/5000). LBMT was estimated to not be cost effective to CDT at the $120,000/QALY WTP threshold in 1.8% (92/5000) of simulations, of which: LBMT was more costly and less effective in 0.6% (29/5000), LBMT was less costly and less effective in 0.4% (21/5,000), and LBMT was more costly and more effective in 0.8% (42/5000).

**Figure 3:**
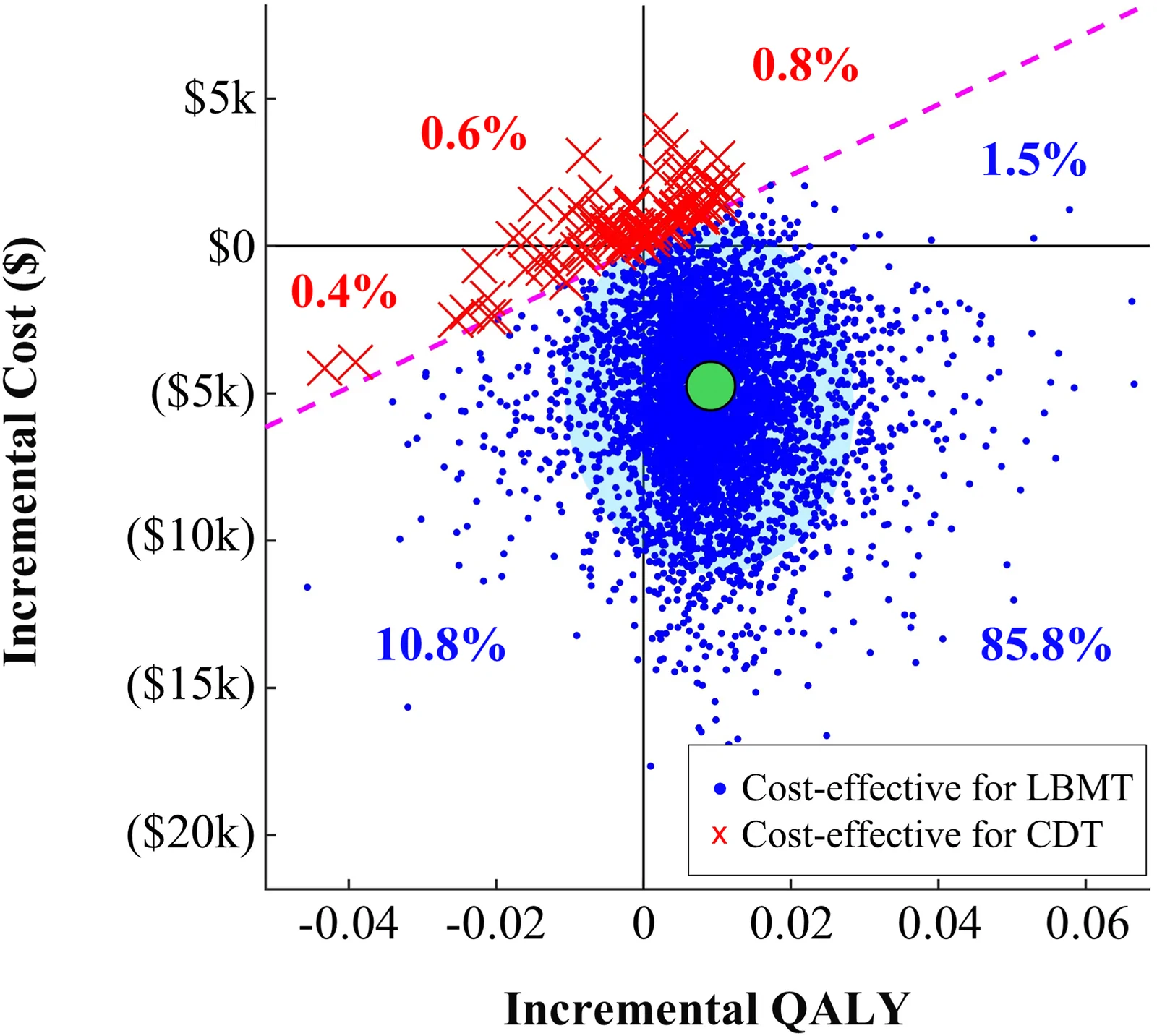
**Probabilistic sensitivity analysis.** Scatter plot of estimated joint density of incremental costs and quality-adjusted life years (QALY) comparing large-bore mechanical thrombectomy (LBMT) and catheter-directed thrombolysis (CDT) by Monte Carlo simulation. The base case result is displayed as a green circle. Evaluations where LBMT or CDT were cost effective are represented by blue dots and red x’s, respectively. The underlying light blue ellipse represents the region containing 95% of simulated results. The willingness-to-pay threshold of $120,000/QALY is represented by the pink dashed line. Percentages indicate the proportion of results within the overlain sector as defined by the standard axes and the willingness-to-pay threshold, with blue and red percentages representing results that are cost effective for LBMT or cost effective for CDT, respectively.

### CDT device cost scenarios

Scenario analyses evaluating extreme distributions of CDT device costs are presented in Table 4. These scenarios assessed the high (ie, assuming all CDT with ultrasound-facilitated CDT) and low (ie, assuming all CDT with standard side-hole catheter) cost assumptions. The results demonstrate a positive NMB (range, $2216-$7725) for LBMT regardless of the assumed distribution of CDT device utilization.

**Table 4:** Discounted cost-effectiveness of LBMT vs CDT for scenario analyses

	LBMT	CDT	Difference	Result
Scenario: Low CDT device cost estimation, lifetime horizon (assuming all CDT with standard side-hole device)				
Lifetime costs, $	68,950	70,071	(1120)	–
QALY	6.374	6.364	0.009	LBMT is cost-saving
LY	7.497	7.496	0.000	–
NMB for LBMT,^a^ $	–	–	–	2216
Scenario: High CDT device cost estimation, lifetime horizon (assuming all CDT with US-CDT device)				
Lifetime costs, $	68,950	75,580	(6630)	–
QALY	6.374	6.364	0.009	LBMT is cost-saving
LY	7.497	7.496	0.000	–
NMB for LBMT,^a^ $	–	–	–	7725
Scenario: Device-specific CTEPH rates, lifetime horizon (CTEPH rate at 5 y; LBMT = 1.81%, CDT = 3.45%)				
Lifetime costs, $	66,855	76,584	(9729)	–
QALY	6.374	6.364	0.011	LBMT is cost-saving
LY	7.532	7.448	0.085	–
NMB for LBMT,^a^ $	–	–	–	10,990

Values are discrete model outputs.

Values in parentheses indicate a negative number.

CDT, catheter-directed thrombolysis; CTEPH, chronic thromboembolic pulmonary hypertension; LBMT, large-bore mechanical thrombectomy; LY, life year; NMB, net monetary benefit; QALY, quality-adjusted life year; US-CDT, ultrasound-facilitated catheter-directed thrombolysis.

a Assuming <$120,000/QALY willingness-to-pay threshold.

### Device-specific CTEPH scenario

A scenario analysis evaluating device-specific CTEPH rates is reported in Table 4. This scenario implemented 5-year CTEPH rates sourced from the single eligible study identified from the systematic review, which reported rates of 1.8% and 3.5% for LBMT and CDT, respectively.^33^ The results demonstrated additional cost-effectiveness and a larger NMB for LBMT ($10,990).

## Discussion

Drawing upon the PEERLESS RCT and relevant literature, this is, to the best of our knowledge, the first economic analysis evaluating the cost-effectiveness of LBMT vs CDT for intermediate-risk PE from the perspective of the US health care system which is informed by randomized data. The results indicate that LBMT using the FlowTriever system is likely to impart economic benefit when compared to CDT. Despite greater initial device and procedure costs, LBMT resulted in lower lifetime costs because of reduced ICU and hospital stay requirements, lower thrombolytic use, and fewer 30-day readmissions, alongside a marginal cost reduction from fewer clinical deteriorations or bailouts. Further, sensitivity analyses indicate that these findings are robust to parameter uncertainty and cost variation attributable to CDT device selection or device-specific rates for CTEPH. These results suggest that LBMT may offer meaningful value compared to CDT, driven primarily by short-term cost savings rather than substantial long-term QALY gains.

The PEERLESS RCT reported that in-hospital clinical deterioration and/or therapy escalation events and postprocedural ICU utilization occurred significantly less frequently for patients receiving LBMT compared to CDT.^3^ Furthermore, the specific deterioration events reported in the LBMT group were less costly to manage in the present model. Additionally, patients receiving LBMT had shorter postprocedural ICU LOS, shorter total and postprocedural hospital LOS, and fewer all-cause readmissions within 30 days. Implementing these data in the present cost-effectiveness model projected a positive lifetime NMB of $5850 with LBMT at the WTP threshold of $120,000/QALY and economic dominance.^10^ Sensitivity analyses reveal that although the cost of the ICU stay, cost of the LBMT device, cost of thrombolytics, length of hospital stay, and rate of readmissions had the largest impact on results, LBMT remained economically favorable, maintaining a positive NMB, in all univariate scenarios and most multivariate simulations. This suggests robust cost-effectiveness across anticipated parameter ranges. Therefore, this study suggests with a high level of certainty that, when compared to CDT, it is likely that LBMT in intermediate-risk PE patients represents a cost effective treatment from the US health care system perspective.^10^

Though a unique strength of the PE management phase of this analysis is its underpinning by high-quality randomized data, it is worth considering additional real-world treatment practices that may not have been captured because of the characteristics of the population enrolled in the PEERLESS RCT. Although the study reported no difference in major bleeding rates between LBMT and CDT, it is important to understand that these conclusions were drawn from a patient cohort with low bleeding risk (ie, 4% of randomized patients had a contraindication to thrombolytics compared to a rate of 25%-32% in prior real-world studies).^3^^,^^4^^,^^28^^,^^39^^,^^40^ A recent retrospective nonrandomized analysis studying LBMT vs CDT in patients with acute PE with higher bleeding risks (ie, 28% contraindicated to thrombolytics) found that LBMT imparted reduced rates of hemodynamic deterioration, major bleeding, and ICH compared to CDT.^28^ Such findings align with results from a meta-analysis that demonstrated elevated bleeding rates following CDT.^6^ As such, the present results may represent a conservative estimate of the economic advantages of LBMT when considering the characteristics of the PEERLESS RCT study population and how these may have differed from real-world patient presentation.

Differences in postprocedural care requirements during the PE management phase also heavily influenced model outcomes. The reduced stay requirements observed with LBMT were a major contributor to the difference in costs between groups. In the PEERLESS RCT, patients treated with LBMT had a lower postprocedural ICU admission rate (ie, 41.6% for LBMT vs 98.6% for CDT), a shorter duration of postprocedure ICU stay (14.2 ± 25.4 hours for LBMT vs 39.3 ± 28.0 hours for CDT), and shorter duration of postprocedure hospital stay (3.2 ± 2.7 days for LBMT vs 4.0 ± 3.7 days for CDT).^3^ Although some of these observed differences in LOS may have been driven by local protocols dictating ICU monitoring during and following CDT treatment, it is also possible that these outcomes were influenced by the reduced need for high-acuity care, lower rate of clinical deterioration and/or bailout events, and earlier signals of patient recovery following LBMT treatment.^3^ In addition, the rates of readmission at 30 days postprocedure were lower for LBMT compared to CDT (3.2% vs 7.9%).^3^ Although ICU stay and readmissions represent acute events, they are particularly relevant to cost-effectiveness estimation as they are relatively large cost drivers and impact hospital resource availability.

Additionally, to ensure that estimates from the long-term care phase were not biased by the initial treatment, the base case analysis applied equal rates of complications for both treatment groups beyond the 30-day PEERLESS trial window. This approach isolates the impact of short-term events on the cost-effectiveness results and may, therefore, underestimate the potential long-term value of intervention. To explore this, a scenario analysis implemented device-specific CTEPH rates sourced from a large, propensity score–matched analysis of real-world evidence that reported a numerically lower rate of CTEPH for LBMT patients compared to CDT patients over the 5-year study period, although this result was not statistically significant.^33^ This analysis demonstrates that in a scenario consistent with this large study in which LBMT is associated with a lower incidence of CTEPH compared to CDT, the NMB for LBMT would increase further, underscoring the importance of long-term follow-up in ongoing and future randomized studies.

Several limitations of this analysis warrant consideration. First, this study follows standard health economic practices by adopting a lifetime horizon; however, we acknowledge the inherent limitations of extrapolating beyond available follow-up data. In particular, factors contributing to long-term costs and health states were derived from published literature rather than from the randomized study. Methods implemented to project long-term costs, health status, and complications were similar to those used in previous cost-effectiveness analyses, and our assumptions were intentionally made to be conservative where possible.^18^^,^^19^^,^^33^ Second, and similarly, parameters describing initial PE management and follow-up were sourced from a single RCT that studied mainly low bleeding risk patients. As such, these results may not directly generalize to a real-world patient population where bleeding risk and contraindication to thrombolytics are generally higher. On a similar note, our model assumes treatment patterns observed within the PEERLESS trial, which may differ in real-world practice based on institution, geography, and patient risk profiles. Third, costs and resource utilization trends frequently vary based on local practices and resource availability, influencing results. Although we attempt to characterize these differences through sensitivity analysis, the results may vary in real-world practice. Fourth, this analysis did not examine differences across patient populations (ie, health equity or health care availability). Future research could assess whether treatment costs and benefits vary by demographic characteristic or socioeconomic factors. Finally, although the study found LBMT with the FlowTriever system to be cost effective, these conclusions may not necessarily extend to other mechanical thrombectomy devices because this was the only LBMT device studied in the PEERLESS RCT. Further prospective, comparative research with longer observation periods in diverse health care settings would strengthen these results.

## Conclusion

In this cost-effectiveness analysis of patients with intermediate-risk PE, LBMT was found to be cost-saving and cost effective compared with CDT, driven by reduced short-term resource utilization and lower total costs. These results suggest that LBMT is an economically attractive strategy from the perspective of the US health care system.

## CRediT authorship contribution statement

**Samuel Horr:** Writing – review & editing, Supervision, Methodology, Conceptualization. **Orestis Pappas:** Writing – review & editing, Methodology. **Ripal T. Gandhi:** Writing – review & editing, Methodology. **Keith Pereira:** Writing – review & editing, Methodology. **Jay Giri:** Writing – review & editing, Supervision, Methodology, Conceptualization. **Sameer J. Khandhar:** Writing – review & editing, Methodology. **Ezana M. Azene:** Writing – review & editing, Methodology. **Joseph Campbell:** Writing – review & editing, Methodology. **Jonathan Belsey:** Writing – review & editing, Validation, Supervision, Methodology, Investigation, Formal analysis, Conceptualization. **Rick Wat:** Writing – original draft, Visualization, Validation, Methodology, Investigation, Data curation, Conceptualization. **Jarrod Collins:** Writing – review & editing, Writing – original draft, Visualization, Validation, Supervision, Project administration, Methodology, Investigation, Formal analysis, Data curation, Conceptualization. **Wissam A. Jaber:** Writing – review & editing, Supervision, Methodology. **Carin F. Gonsalves:** Writing – review & editing, Supervision, Methodology. **Stefan Stortecky:** Writing – review & editing, Supervision, Methodology.

## Declaration of competing interest

Samuel Horr reports consultant fees from Inari Medical, Inquis Medical, Abbott Vascular, and Edwards Lifesciences. Orestis Pappas reports research grant/funding from Inari Medical and consultant fees from Abiomed, Boston Scientific, Inari Medical, Medtronic, and Zoll. Ripal T. Gandhi reports consultant fees from Argon, BD, Boston Scientific, Cook, Cordis, Inari Medical, Medtronic, and Penumbra. Keith Pereira reports having no conflicts relevant to this work. Jay Giri reports research grant/funding from Edwards Lifesciences and Inari Medical; consultant fees from Boston Scientific and Inari Medical; honoraria from Edwards Lifesciences; equity ownership in Endovascular Engineering; and advisory board membership with Boston Scientific. Sameer J. Khandhar reports consultant fees from Inari Medical and Neptune Medical. Ezana M. Azene reports research grant/funding from Endovascular Engineering and Inari Medical; consultant fees from Medtronic and Philips; and honoraria from Philips. Joseph Campbell reports consultant fees from Inari Medical. Jonathan Belsey reports consultant fees from Boston Scientific and Inari Medical. Rick Wat and Jarrod Collins are employees of Inari Medical. Wissam A. Jaber reports research grant/funding from Inari Medical, Medtronic, and Thrombolex; consultant fees from Abbott, Inari Medical, and Medtronic; and advisory board membership with Thrombolex. Carin F. Gonsalves reports research grant/funding from Inari Medical and consultant fees from Inari Medical. Stefan Stortecky reports research grants to the institution from Edwards Lifesciences, Medtronic, Boston Scientific, and Abbott, as well as consultant fees without personal remuneration from Inari Medical.

## Footnotes

To access the supplemental material accompanying this article, visit the online version of the *Journal of the Society for Cardiovascular Angiography & Interventions* at 10.1016/j.jscai.2026.105578.
