# Supplementary material for: Cost-Effectiveness of Large-Bore Mechanical Thrombectomy Versus Catheter-Directed Thrombolysis for Intermediate-Risk Pulmonary Embolism: A PEERLESS Randomized Controlled Trial Post Hoc Analysis
Source: J Soc Cardiovasc Angiogr Interv. 2026 Aug 25;5(10):105578. doi: 10.1016/j.jscai.2026.105578 (PMC13700045; doi:10.1016/j.jscai.2026.105578)
Supplement: Supplementary file 1 [file mmc1.docx]

**Supplemental Materials**

**Supplemental Methods: Determination of medical care costs**	Page 2

Cost framework	Page 2

PE management phase	Page 2

PE management phase – procedure costs	Page 2

PE management phase – LBMT-specific costs	Page 3

PE management phase – CDT-specific costs	Page 3

PE management phase – Bailout-specific costs	Page 3

Long-term care phase	Page 4

**Supplemental Methods: Consolidated health economic evaluation reporting standards**	Page 5

Supplemental Table 1. CHEERS 2022 checklist	Page 5

**Supplemental Methods: Criteria for health economic quality evaluation tool**	Page 7

Supplemental Table 2. CHEQUE tool: methods quality	Page 7

Supplemental Table 3. CHEQUE tool: reporting quality	Page 10

**Supplemental Methods: Impact inventory**	Page 12

Supplemental Table 4. Impact inventory table	Page 12

**Supplemental Methods: Model parameterization**	Page 14

Supplemental Table 5. Details of CDT device selection from PE management phase	Page 14

Supplemental Table 6. Health state probabilities for PE management phase	Page 14

Supplemental Table 7. Applied distribution of CDBO events	Page 14

Supplemental Table 8. Health state probabilities for long-term care phase	Page 15

Supplemental Table 9**.** Utilities and disutilities for all care phases	Page 16

Supplemental Table 10. Costs associated with PE management phase health states	Page 17

Supplemental Table 11. Microcosting of treatment in PE management phase	Page 17

Supplemental Table 12. Resource utilization from PE management phase	Page 18

Supplemental Table 13. Costs associated with long-term care phase health states	Page 19

**Supplemental Methods: Systematic literature review of device-specific CTEPH rates**	Page 20

Overview and search structure	Page 20

PRISMA diagram	Page 21

Search results	Page 22

**Supplemental Methods: Model validation**	Page 23

**References**	Page 24

**Supplemental Methods: Determination of medical care costs**

**Cost framework**

The present analysis focuses solely on direct medical costs incurred by the hospital, excluding indirect costs (e.g., lost productivity).^13^ Costs from years before 2024 were converted to 2024 US dollars using the medical care Consumer Price Index.^36^ Microcosting values were derived from a review of the published literature, with targeted supplementation from the PREMIER Healthcare Database, a large, generalizable US hospital-based, service-level, all-payer database containing more than 172 million inpatient visits.^35^

Costs were categorized as fixed, variable, or center-specific, consistent with previous cost-comparison publications.^12, 13^ Fixed costs represent standard expenses incurred for all patients undergoing the procedure. Variable costs depend on individual patient outcomes and additional resources required during treatment. Center-specific costs reflect variations in treatment protocols across institutions and were incorporated into sensitivity analyses to assess the robustness of the findings.^13^

**PE management phase**

Costs for the index hospitalization and follow-up period (i.e., the PE management phase) were assessed using a combination of resource-based accounting, event-based methods, and hospital billing data,^13, 35^ consistent with previous economic analyses.^41-44^ The resource-based accounting approach is implemented to determine procedure-related costs and is largely derived from Tran et al. (2024).^13^ The event-based approach for determining costs associated adverse outcomes following the index procedure and resource utilization during the index hospitalization is based on the methodology of Noman et al. (2024).^12^

**PE management phase – procedure costs**

The cost of catheterization lab for the index procedure was determined by standard, “bottoms-up” cost-account methodology.^41-44^ The cost of cardiac catheterization lab for a procedure was estimated on the basis of the average per-minute operating room cost, adjusted according to actual procedure duration.^3, 12, 13^ Device-specific costs were derived from published literature that referenced manufacturer-provided pricing, ensuring broader generalizability.^11, 12^ Detailed resource utilization and cost for items such as catheters, sheaths, guiding catheters, guidewires, and other devices were identified based on published literature.^3, 13^ PREMIER and other published sources provided supplementary cost data where needed. Imaging costs included standardized protocols for both LBMT and CDT arms, comprising chest CT scan, lower extremity venous doppler ultrasound, and echocardiogram.^13^ CDT procedures additionally assumed follow-up chest radiography and selective echocardiogram testing as clinically indicated.^13^ Laboratory costs comprised routine labs plus daily hematologic, metabolic, and coagulation panels.^13^ CDT procedures incurred additional costs for follow-up venous blood gas analysis.^13^ Pharmaceutical costs included pre- and post-procedural unfractionated heparin, along with intraprocedural medications (sedation, local anesthesia, additional anticoagulation).^13^ Post-discharge medications were not considered. Secondary costs associated with additional peri-procedural resource utilization (i.e., total hospital stay, post-procedure ICU stay, and 30-day readmission) were calculated following treatment based on PEERLESS trial data.^3^

**PE management phase – LBMT-specific costs**

The costs associated with LBMT treatment were categorized as follows:

(1) Fixed cost variables included standard components for LBMT that every patient received: facility costs (imaging, cardiac catheterization lab); procedural device (FlowTriever); access and support equipment (Intri24™ sheath or Gore® DrySeal sheath, 7FR sheath, micropuncture kit, Amplatz Super Stiff™ wire, Soren dilator kit); catheterization equipment (balloon-tipped right heart catheter); procedural supplies (catheterization sterile packet, contrast materials, ultrasound probe cover); and drugs required for the catheterization lab.^12, 13^
(2) Variable costs included patient-specific factors such as length of stay (hospital and intensive care unit [ICU]), medications (intraprocedural pharmaceuticals, contrast materials), laboratory testing, and post-procedure echocardiograms.^12, 13^
(3) Center-specific costs accounted for variations in procedural protocols, such as the use of different hemostasis methods (e.g., FlowStasis™, mattress suture, or Perclose™) and different catheter types for pulmonary artery angiograms;^13^ these variations were incorporated into sensitivity analyses to test the robustness of the findings.

**PE management phase – CDT-specific costs**

The costs associated with CDT treatment were categorized as follows:

(1) Fixed costs included standard components for all patients: facility costs (imaging, cardiac catheterization lab, post-procedure X-ray); catheterization equipment (Swan Ganz catheter, catheter for pulmonary artery angiogram); access and support equipment (vascular access sheaths, 240 cm exchange 0.035-inch intravascular wire, micropuncture access kit); procedural supplies (catheterization sterile packet, ultrasound probe cover, contrast dye); and drugs required for the catheterization lab.^12, 13^
(2) Variable costs encompassed patient-specific factors including length of stay (hospital and ICU), medications (intraprocedural pharmaceuticals), and laboratory testing (including fibrinogen levels).^12, 13^
(3) Center-specific costs included variations in thrombolytic vial sizes (e.g. 50mg or 10mg vial sizes) for sensitivity testing^13^ and the cost of various CDT devices (i.e., ultrasound-facilitated, standard side-hole, standard side-slit, pharmacochemical, gradient side-hole, and >1 device used) according to their use distribution in the PEERLESS RCT.^3^

**PE management phase – Bailout-specific costs**

The costs of secondary procedures for patients requiring bailout therapy due to clinical deterioration were derived using the same methodology as the primary procedures, supplemented by published literature. To ensure methodological consistency in cost calculations and avoid potential bias from outlier scenarios, our analysis considered the initial bailout intervention observed in the PEERLESS trial. The primary observed bailout pathways were LBMT for CDT patients and systemic thrombolysis for LBMT patients.^3^ The utilization rates of these bailout procedures were based on the PEERLESS trial data.^3^ Secondary procedure costs incorporated additional resource use, including incremental facility costs (procedure time), post-procedure monitoring (X-ray, laboratory tests), length of stay (hospital and ICU), thrombolytic medication costs (for LBMT requiring systemic thrombolysis bailout), and thrombectomy device costs (for CDT requiring LBMT bailout).

**Long-term care phase**

Costs for the long-term care phase were either implemented as treatment costs (e.g., continued anticoagulation management) or using event-based methods for adverse outcomes. Event-based costs were either implemented when an event occurred or as a recurrent maintenance cost to account for long-term rehabilitation and follow-up.

**Supplemental Methods: Consolidated health economic evaluation reporting standards**

# **Supplemental Table 1.** CHEERS 2022 checklist

| **Topic** | **No.** | **Item** | **Location where item is reported** |
| --- | --- | --- | --- |
| **Title** |  |  |  |
|  | 1 | Identify the study as an economic evaluation and specify the interventions being compared. | Title |
| **Abstract** |  |  |  |
|  | 2 | Provide a structured summary that highlights context, key methods, results, and alternative analyses. | Abstract |
| **Introduction** |  |  |  |
| **Background and objectives** | 3 | Give the context for the study, the study question, and its practical relevance for decision making in policy or practice. | Background |
| **Methods** |  |  |  |
| **Health economic analysis plan** | 4 | Indicate whether a health economic analysis plan was developed and where available. | NA |
| **Study population** | 5 | Describe characteristics of the study population (such as age range, demographics, socioeconomic, or clinical characteristics). | Methods – PE management phase |
| **Setting and location** | 6 | Provide relevant contextual information that may influence findings. | Methods – model overview |
| **Comparators** | 7 | Describe the interventions or strategies being compared and why chosen. | Methods |
| **Perspective** | 8 | State the perspective(s) adopted by the study and why chosen. | Methods |
| **Time horizon** | 9 | State the time horizon for the study and why appropriate. | Methods – model overview |
| **Discount rate** | 10 | Report the discount rate(s) and reason chosen. | Methods – cost parameters |
| **Selection of outcomes** | 11 | Describe what outcomes were used as the measure(s) of benefit(s) and harm(s). | Methods – analytic endpoints |
| **Measurement of outcomes** | 12 | Describe how outcomes used to capture benefit(s) and harm(s) were measured. | Methods – PE management phase  Methods – long-term care phase |
| **Valuation of outcomes** | 13 | Describe the population and methods used to measure and value outcomes. | Methods – analytic endpoints |
| **Measurement and valuation of resources and costs** | 14 | Describe how costs were valued. | Methods – analytic endpoints  Supplemental Methods: Determination of medical care costs |
| **Currency, price date, and conversion** | 15 | Report the dates of the estimated resource quantities and unit costs, plus the currency and year of conversion. | Methods – cost parameters |
| **Rationale and description of model** | 16 | If modelling is used, describe in detail and why used. Report if the model is publicly available and where it can be accessed. | Methods – model overview  Methods – PE management phase  Methods – long-term care phase |
| **Analytics and assumptions** | 17 | Describe any methods for analysing or statistically transforming data, any extrapolation methods, and approaches for validating any model used. | Methods – analytic endpoints  Methods – sensitivity analysis |
| **Characterising heterogeneity** | 18 | Describe any methods used for estimating how the results of the study vary for subgroups. | NA |
| **Characterising distributional effects** | 19 | Describe how impacts are distributed across different individuals or adjustments made to reflect priority populations. | NA |
| **Characterising uncertainty** | 20 | Describe methods to characterise any sources of uncertainty in the analysis. | Methods – sensitivity analysis |
| **Approach to engagement with patients and others affected by the study** | 21 | Describe any approaches to engage patients or service recipients, the general public, communities, or stakeholders (such as clinicians or payers) in the design of the study. | NA |
| **Results** |  |  |  |
| **Study parameters** | 22 | Report all analytic inputs (such as values, ranges, references) including uncertainty or distributional assumptions. | Supplemental Methods: Model parameterization |
| **Summary of main results** | 23 | Report the mean values for the main categories of costs and outcomes of interest and summarise them in the most appropriate overall measure. | Results  Results – PE management phase  Results – long-term care phase |
| **Effect of uncertainty** | 24 | Describe how uncertainty about analytic judgments, inputs, or projections affect findings. Report the effect of choice of discount rate and time horizon, if applicable. | Results – sensitivity analyses |
| **Effect of engagement with patients and others affected by the study** | 25 | Report on any difference patient/service recipient, general public, community, or stakeholder involvement made to the approach or findings of the study | NA |
| **Discussion** |  |  |  |
| **Study findings, limitations, generalisability, and current knowledge** | 26 | Report key findings, limitations, ethical or equity considerations not captured, and how these could affect patients, policy, or practice. | Discussion |
| **Other relevant information** |  |  |  |
| **Source of funding** | 27 | Describe how the study was funded and any role of the funder in the identification, design, conduct, and reporting of the analysis | Sources of funding |
| **Conflicts of interest** | 28 | Report authors conflicts of interest according to journal or International Committee of Medical Journal Editors requirements. | Disclosures |

**Supplemental Methods: Criteria for health economic quality evaluation tool**

# **Supplemental Table 2.** CHEQUE tool: methods quality

| **[Domain]**  **Attribute** | **Rounded importance score** | **Scoring weight assessment∗ (yes, somewhat, no, or N/A)** | **Comments** | **Final score** |
| --- | --- | --- | --- | --- |
| **[Decision problem and scope]** |  |  |  |  |
| M1. The analysis answers an important question for decision making. | 5 | Yes | The study addresses whether LBMT is cost-effective compared to CDT for intermediate-risk PE, an important question for clinical and health system decision makers. | 5 |
| M2. The study objective (decision problem) is measurable. | 6 | Yes | The objective of study is measurable in terms of costs, QALYs, LYs, ICERs, and NMB. | 6 |
| **[Intervention and comparator(s)]** |  |  |  |  |
| M3. The comparator(s) is/are the best possible option that appropriately measures the opportunity cost of using the new treatment. | 4 | Somewhat | CDT is the main alternative intervention in clinical practice, although anticoagulation alone is not modelled. Including anticoagulation alone would require assumptions unsupported by RCT data at the time of publication. | 2 |
| **[Perspective]** |  |  |  |  |
| M4. The analytic perspective(s) is/are appropriate to answer the research question posed. | 4 | Yes | U.S. healthcare system perspective is appropriate for payer decision-making. | 4 |
| **[Population]** |  |  |  |  |
| M5. The scope of the study encompasses all populations affected by the intervention. | 1 | Somewhat | The scope of study is limited to intermediate-risk PE only, excluding high-risk PE. Including high-risk PE population would require assumptions unsupported by RCT data at the time of publication. | 0.5 |
| **[Outcome measures]** |  |  |  |  |
| M6. Health outcomes are measured in health metrics that aggregate survival and health-related quality of life or disability (eg, QALY or DALY). | 3 | Yes | Health outcomes are measured in QALYs and LYs for each treatment arm. | 3 |
| **[Time horizon]** |  |  |  |  |
| M7. The analytic time horizon is sufficiently long enough to reflect all important differences between intervention(s) and comparator(s). | 4 | Yes | The analytic time horizon is over lifetime period, reflecting long-term chronic complications of PE, such as recurrent VTE, bleeding, and CTEPH. | 4 |
| **[Discounting]** |  |  |  |  |
| M8. Costs and health effects that occur in the future are discounted to their present value using a recommended discount rate. | 2 | Yes | Costs and health effects are discounted at 3% rate. | 2 |
| **[Modeling]** |  |  |  |  |
| M9. The chosen model type is appropriate to address study questions. | 6 | Yes | The chosen model type (i.e., combined decision tree for acute phase and Markov model for long-term outcomes) is appropriate to address the decision problem. | 6 |
| M10. The structure of the model reflects the underlying health condition and the impact of the interventions. | 8 | Yes | The structure of the model includes acute and long-term phases to reflect the underlying health condition and impact of interventions. The acute phase includes acute complications, ICU stay, readmissions, and the long-term phase include recurrent VTE, bleeding, and CTEPH. | 8 |
| M11. Modeling assumptions are reasonable, given the underlying data. | 5 | Yes | The modelling assumptions are reasonable, given the underlying data, which is limited to RCT evidence for acute phase outcomes. | 5 |
| M12. The need for extrapolation or integrating multiple data sources is considered. | 3 | Yes | The model conservatively assumes equal long-term event rates across arms for the lifetime extrapolation using data from multiple sources (i.e., systematic review, meta-analysis, RCTs) | 3 |
| M13. Model validation is conducted, including an assessment of the model structure, assumptions, data, and results. | 8 | Yes | Model validation is performed, including an assessment of model structure, assumptions, data, and results, and presented in the Supplementary materials | 8 |
| **[Data inputs and evidence synthesis]** |  |  |  |  |
| M14. A “best available evidence” approach is used to select data sources for model parameters (eg, conducted or references systematic reviews/meta-analyses). | 6 | Yes | The clinical input for acute phase is based on PEERLESS RCT data, and for long-term phase is based on systematic literature review for CTEPH and AMPLIFY-EXT RCTs for bleeding and recurrent VTE rates. Cost input based on large all-payer hospital administrative database (PHD). | 6 |
| M15. Data inputs are generated by appropriate statistical and epidemiological techniques. | 5 | Yes | Clinical inputs for acute and long-term phases are derived from their respective sources using appropriate statistical and epidemiological techniques. | 5 |
| M16. The quality of the data, including sources of bias, is assessed appropriately. | 6 | Somewhat | Data quality ensured through use of RCT and systematic reviews; formal bias assessment not conducted but limitations and potential bias acknowledged. | 3 |
| **[Consequences]** |  |  |  |  |
| M17. Major consequences affected by the choice of interventions being compared are identified. | 5 | Yes | Major consequences affected by choice of intervention, including acute complications (i.e., mortality, bleeding, clinical deterioration and/or escalation to bailout), long-term complications (i.e., CTEPH, recurrent VTE, and bleeding) and healthcare resource utilization (i.e., ICU stay, LOS, readmissions) are compared between treatment arms. | 5 |
| **[Utilities (preference measures)]** |  |  |  |  |
| M18. Health preferences reflect those of the jurisdiction(s) of interest (as specified in the decision problem). | 2 | Yes | Health preferences and utility weights reflect individuals with intermediate-risk PE in the United States. | 2 |
| **[Costs and resource use]** |  |  |  |  |
| M19. Resource use that is nontrivial in magnitude is included in the reference case analysis. | 2 | Yes | Resource use of nontrivial magnitude, such as device cost, procedure cost, ICU and hospital stay, readmissions, long-term complications, and other costs are included in reference analysis. | 2 |
| **[Analysis]** |  |  |  |  |
| M20. Incremental analyses are conducted (ie, the additional costs generated by one alternative over another are compared with the additional effects generated). | 4 | Yes | Incremental costs, incremental QALYs, incremental LYs, and NMB are reported. It is generally not recommended to explicitly report negative ICERs in dominant scenarios. | 4 |
| M21. ICERs are obtained by comparing each intervention with the next most effective option after eliminating dominated options. | 3 | Yes | Two options are compared and LBMT is the dominant strategy. The ICER is not explicitly reported due to dominance of LBMT over CDT. | 3 |
| M22. Probabilistic sensitivity analysis is conducted to account for uncertainty in input parameters simultaneously. | 3 | Yes | Probabilistic sensitivity analysis is conducted to account for uncertainty in inputs simultaneously, with 5,000 simulations and scatter plot reported. | 3 |
| M23. Alternative modeling choices and assumptions (structural uncertainty) are explored through additional sensitivity analysis (ie, scenario analysis). | 4 | Yes | Multiple scenario analysis, including scenarios for CDT device cost and device-specific CTEPH rates were performed and reported. | 4 |
| **[Equity considerations]** |  |  |  |  |
| M24. Relevant equity or distributional considerations are taken into account. | 1 | No | Equity and distributional considerations were not explicitly modelled but are noted as a study limitation. | 0 |
| **[Total]** | **100** |  |  | **93.5** |
| *Scoring weight: “yes” = 1.0; “somewhat” = 0.5; “no” = 0.0. | | | | |

# **Supplemental Table 3.** CHEQUE tool: reporting quality

| **[Domain] Attribute** | **Rounded importance score** | **Scoring weight assessment∗ (yes, somewhat, no, or N/A)** | **Comments** | **Final score** |
| --- | --- | --- | --- | --- |
| **[Decision problem and scope]** |  |  |  |  |
| R1. The study objectives (or decision problems) are clearly stated. | 7 | Yes | The study objective is clearly stated in abstract and background | 7 |
| **[Intervention and comparator(s)]** |  |  |  |  |
| R2. All aspects of the interventions that may affect their cost-effectiveness are clearly defined (eg, frequency of delivery, setting of delivery, and specific technologies used). | 7 | Yes | All aspects of the intervention are defined in the methods section. The details are reported in the supplementary materials, including frequency of delivery, setting of care, and technologies used. | 7 |
| R3. The comparator(s) is/are clearly stated. | 6 | Yes | The comparator is specified in the methods section. | 6 |
| **[Perspective]** |  |  |  |  |
| R4. The analytic perspective(s) is/are clearly stated. | 4 | Yes | U.S. healthcare system perspective reported in the methods section. | 4 |
| **[Population]** |  |  |  |  |
| R5. The target population is clearly stated. | 4 | Yes | The target population (intermediate-risk PE) is stated in methods section – PE management phase | 4 |
| **[Outcome measures]** |  |  |  |  |
| R6. Primary outcome measures are clearly stated. | 5 | Yes | The primary outcome measures (costs, QALYs, LYs, ICER, NMB) are stated in the methods section – analytic endpoints | 5 |
| R7. ICERs are reported. | 4 | Yes | Dominance is reported. In cases of dominance, ICER calculation is not recommended due to the difficulty of interpreting negative values. We therefore use the term "dominant" rather than explicitly reporting a numerical ICER, consistent with standard practice in health economic evaluation. | 4 |
| **[Time Horizon]** |  |  |  |  |
| R8. The analytic time horizon is clearly stated. | 3 | Yes | Lifetime horizon specified in methods section, model overview | 3 |
| **[Discounting]** |  |  |  |  |
| R9. The discount rate is clearly stated. | 2 | Yes | 3% per year for costs and outcomes is specified in methods section, cost parameters | 2 |
| **[Modeling]** |  |  |  |  |
| R10. The type of model used is clearly stated. | 4 | Yes | The type of model (combined decision tree + Markov model) is specified in methods section, model overview | 4 |
| R11. Justification of modeling choices and assumptions is provided. | 8 | Yes | The justification of modeling choices and assumptions are reported in the methods section and supplemental methods: determination of medical care costs. | 8 |
| R12. Model descriptions are detailed enough to allow for replication. | 9 | Yes | Detailed descriptions of the model are reported in the methods section and supplemental methods to enable replication. | 9 |
| R13. The description of how the model was validated is provided. | 3 | Yes | Model validation is described in methods section and supplemental methods: model validation | 3 |
| R14. The software used to develop the model is clearly stated. | 5 | Yes | The software (Excel v2503) is reported in methods section | 5 |
| **[Data inputs and evidence synthesis]** |  |  |  |  |
| R15. All data sources are clearly referenced. | 5 | Yes | All data sources are clearly referenced in the manuscript and supplemental methods | 5 |
| **[Consequences]** |  |  |  |  |
| R16. Comprehensive identification of potential consequences is summarized (eg, using an Impact Inventory table in Second Panel’s report). | 2 | Yes | An impact inventory table is provided in supplemental methods: impact inventory to summarize the inclusion/exclusion of specific value criteria. | 2 |
| **[Utilities (preference measures)]** |  |  |  |  |
| R17. Sources for the utility weights are clearly stated. | 3 | Yes | Sources for utility weights are cited in the manuscript and supplement materials | 3 |
| **[Costs and resource use]** |  |  |  |  |
| R18. Quantities of resources are reported separately from the prices (unit costs) of those resources. | 2 | Yes | Supplementary tables report the quantities of resources used separately from unit prices. | 2 |
| **[Analysis]** |  |  |  |  |
| R19. The approach to secondary analyses (eg, sensitivity, scenario, or subgroup analysis) is sufficiently described. | 3 | Yes | Sensitivity and scenario analysis explained in methods section, sensitivity analyses | 3 |
| **[Equity considerations]** |  |  |  |  |
| R20. Discussion section includes a description of any significant ethical implications of the CEA results. | 1 | Somewhat | The need to assess how costs and QALYs are distributed across patient subgroups is mentioned in the discussion limitations | 0.5 |
| **[Transparency and reporting]** |  |  |  |  |
| R21. Results are presented in a disaggregated format for transparency. | 3 | Yes | Results are presented in disaggregated format for transparency in the results section (Tables 1-4) | 3 |
| R22. The relevance of study results to specific decision problems is discussed. | 3 | Yes | Discussion ties findings to U.S. healthcare system perspective and ACC/AHA thresholds. | 3 |
| R23. Implications of uncertainty for decision making, including the need for future research, are explored. | 3 | Yes | Probabilistic sensitivity analyses, tornado diagrams, and scenario analyses are discussed, including need for future research. | 3 |
| R24. Potential bias and limitations are discussed. | 4 | Yes | Limitations section covers generalizability, bleeding risk, and cost variation. | 4 |
| **[Total]** | 100 |  |  | 99.5 |
| *Scoring weight: “yes” = 1.0; “somewhat” = 0.5; “no” = 0.0. | | | | |

**Supplemental Methods: Impact inventory**

The impact inventory, recommended by the Second Panel on Cost-Effectiveness in Health and Medicine, is a standardized table listing the inclusion and exclusion of specific value criteria to enable comparison across health economic studies.

# **Supplemental Table 4.** Impact inventory table

| Sector | Type of Impact (list category within each sector with unit of measure if relevant) | Included in This Reference Case Analysis From... Perspective? | | Notes on Sources of Evidence |
| --- | --- | --- | --- | --- |
|  |  | Healthcare Sector | Societal |  |
| Formal Health Care Sector | |  |  |  |
| Health | Health outcomes (effects) |  |  |  |
|  | Longevity effects | ✓ | ✗ | Mortality modeled from PEERLESS RCT. |
|  | Health-related quality-of-life effects | ✓ | ✗ | QALYs from decision tree + Markov model. Utility weights from literature. |
|  | Other health effects (eg, adverse events and secondary transmissions of infections) | ✓ | ✗ | Bleeding, bailout events from PEERLESS RCT. |
|  | Medical costs |  |  |  |
|  | Paid for by third-party payers | ✓ | ✗ | Device, ICU, hospital stay, thrombolytic, readmission costs. Costs from literature and Premier database. |
|  | Paid for by patients out-of-pocket | ✗ | ✗ |  |
|  | Future related medical costs (payers and patients) | ✓ | ✗ | Long-term cost of complications and management from systematic review or extrapolation of RCT evidence. |
|  | Future unrelated medical costs (payers and patients) | ✗ | ✗ |  |
| Informal Health Care Sector | |  |  |  |
| Health | Patient-time costs | NA | ✗ |  |
|  | Unpaid caregiver-time costs | NA | ✗ |  |
|  | Transportation costs | NA | ✗ |  |
| Non-Health Care Sectors (with examples of possible items) | |  |  |  |
| Productivity | Labor market earnings lost | NA | ✗ |  |
|  | Cost of unpaid lost productivity due to illness | NA | ✗ |  |
|  | Cost of uncompensated household production | NA | ✗ |  |
| Consumption | Future consumption unrelated to health | NA | ✗ |  |
| Social Services | Cost of social services as part of intervention | NA | ✗ |  |
| Legal/Criminal Justice | Number of crimes related to intervention | NA | ✗ |  |
|  | Cost of crimes related to intervention | NA | ✗ |  |
| Education | Impact of intervention on educational achievement of population | NA | ✗ |  |
| Housing | Cost of intervention on home improvements (eg, removing lead paint) | NA | ✗ |  |
| Environment | Production of toxic waste pollution by intervention | NA | ✗ |  |
| Other (specify) | Other impacts | NA | ✗ |  |

**Supplemental Methods: Model parameterization**

| **Supplemental Table 5.** Details of CDT device selection from PE management phase | | | | | |
| --- | --- | --- | --- | --- | --- |
| **Variable** | **Base** | **Low** | **High** | **Distribution** | **Source** |
| Ultrasound-facilitated CDT | 59.8% | 53.7% | 65.6% | Dirichlet | ^3^ |
| Standard side-hole CDT | 23.2% | 18.3% | 28.6% | Dirichlet | ^3^ |
| Standard side-slit CDT | 8.7% | 5.7% | 12.7% | Dirichlet | ^3^ |
| Pharmacomechanical CDT | 4.3% | 2.3% | 7.5% | Dirichlet | ^3^ |
| Gradient side-hole CDT | 1.1% | 0.2% | 3.1% | Dirichlet | ^3^ |
| Multiple CDT devices used | 2.9% | 1.3% | 5.6% | Dirichlet | ^3^ |
| **Total** | 100.0% |  |  |  |  |
| CDT = catheter directed thrombolysis; PE = pulmonary embolism. | | | | | |

| **Supplemental Table 6.** Health state probabilities for PE management phase | | | | |
| --- | --- | --- | --- | --- |
| **Variable** | **LBMT N=274** | **CDT**  **N=276** | **Distribution** | **Source** |
| **All-cause mortality*** | **0.0% (0)** | **0.4% (1)** | Dirichlet | ^3,†^ |
| **Major bleed*** |  |  |  |  |
| IC bleed, nonfatal | **0.7% (2)** | **0.4% (1)** | Dirichlet | ^3,†^ |
| EC bleed, nonfatal | **6.2% (17)** | **6.2% (17)** | Dirichlet | ^3,†^ |
| **CDBO*** | **1.5% (4)** | **4.3% (12)** | Dirichlet | ^3,†^ |
| **CRNM bleed*** | **2.6% (7)** | **2.5% (7)** | Dirichlet | ^3,†^ |
| **No complications*** | **89.1% (244)** | **86.2% (238)** | Dirichlet | Derived^,†^ |
| **Total** | **100% (274)** | **100% (276)** |  |  |
| Proportions listed as % (n). ^*^Measured at the sooner of hospital discharge or 7 days following the procedure. ^†^Event probabilities were assumed based on each patient’s highest severity event in order of all-cause mortality, major IC bleed, major EC bleed, CDBO, and CRNM bleed, with the remainder captured as no complications. Health outcomes are mutually exclusive at the subject level. PE = pulmonary embolism; LBMT = large bore mechanical thrombectomy; CDT = catheter directed thrombolysis; IC = intracranial; EC = extracranial; CDBO = clinical deterioration and/or bailout; CRNM = clinically relevant non-major. | | | | |

| **Supplemental Table 7.** Applied distribution of CDBO events | | | | |
| --- | --- | --- | --- | --- |
| **Event type** | **LBMT**  **N=5** | **CDT N=15** | **Source** |  |
| CD, cardiac arrest | 0.0% | 6.7% | ^3^ |  |
| CD, high grade AV block | 0.0% | 6.7% | ^3^ |  |
| CD, respiratory failure | 0.0% | 20.0% | ^3^ |  |
| CD, increased oxygen requirement | 20.0% | 0.0% | ^3^ |  |
| CD, hypotension | 60.0% | 26.7% | ^3^ |  |
| BO, systemic thrombolysis | 20.0% | 0.0% | ^3^ |  |
| BO, LBMT | 0.0% | 33.3% | ^3^ |  |
| CD, cardiac arrest + BO, systemic thrombolysis | 0.0% | 6.7% | ^3^ |  |
| **Total** | **100.00%** | **100.00%** |  |  |
| The applied distribution of CDBO events is representative of all subjects experiencing CDBO events encountered in the PEERLESS trial. LBMT = large bore mechanical thrombectomy; CDT = catheter directed thrombolysis; CD = clinical deterioration; AV = atrioventricular; BO = bailout. | | | |  |

| **Supplemental Table 8.** Health state probabilities for long-term care phase | | | | | |  |
| --- | --- | --- | --- | --- | --- | --- |
| **Variable** | **Base** | **Low** | **High** | **Distribution** | **Source** | |
| **Risk of recurrent VTE and VTE-related death** |  |  |  |  |  | |
| 0-3 months | 1.71% | 1.20% | 2.20% | Beta | ^18^ | |
| 3-6 months | 0.48% | 0.22% | 0.75% | Beta | ^18^ | |
| 6-9 months | 2.77% | 1.66% | 3.89% | Beta | ^18^ | |
| 9-12 months | 2.65% | 1.56% | 3.75% | Beta | ^18^ | |
| 12-15 months | 2.17% | 1.18% | 3.16% | Beta | ^18^ | |
| 15-18 months | 1.21% | 0.46% | 1.95% | Beta | ^18^ | |
| Distribution of recurrent VTE events |  |  |  |  |  | |
| On-treatment (AC), 0-18 months |  |  |  |  |  | |
| Recurrent PE | 37.69% |  |  | Dirichlet | ^17^ | |
| Recurrent DVT | 40.77% |  |  | Dirichlet | ^17^ | |
| VTE-related death | 21.54% |  |  | Dirichlet | ^17^ | |
| **Total** | 100.00% |  |  |  |  | |
| Off-treatment (AC), 0-18 months |  |  |  |  |  | |
| Recurrent PE | 24.75% |  |  | Dirichlet | ^17^ | |
| Recurrent DVT | 63.37% |  |  | Dirichlet | ^17^ | |
| VTE-related death | 11.88% |  |  | Dirichlet | ^17^ | |
| **Total** | 100.00% |  |  |  |  | |
| Off-treatment (AC), >18 months |  |  |  |  |  | |
| Recurrent PE | 28.09% |  |  | Dirichlet | ^17^ | |
| Recurrent DVT | 71.91% |  |  | Dirichlet | ^17^ | |
| **Total** | 100.00% |  |  |  |  | |
| **Risk of major bleeding** |  |  |  |  |  | |
| 0-3 months | 0.41% | 0.17% | 0.65% | Beta | ^18^ | |
| 3-6 months | 0.15% | 0.00% | 0.30% | Beta | ^18^ | |
| >6 months, per year | 0.48% | 0.01% | 0.96% | Beta | ^18^ | |
| Additional risk ratio per decade of life | 1.97 | 1.79 | 2.16 | Triangular | ^69^ | |
| Distribution of major bleeding events |  |  |  |  |  | |
| Fatal bleeding event | 13.46% |  |  | Dirichlet | ^18^ | |
| Nonfatal bleeding event | 86.54% |  |  | Dirichlet | ^18^ | |
| **Total** | 100.00% |  |  |  |  | |
| Nonfatal IC bleeding event | 13.97% |  |  | Dirichlet | ^18^ | |
| Nonfatal EC bleeding event | 86.03% |  |  | Dirichlet | ^18^ | |
| **Total** | 100.00% |  |  |  |  | |
| **Risk of CRNM bleeding** |  |  |  |  |  | |
| 0-3 months | 2.65% | 2.04% | 3.26% | Beta | ^18^ | |
| 3-6 months | 1.20% | 0.78% | 1.61% | Beta | ^18^ | |
| >6 months | 2.30% | 1.28% | 3.32% | Beta | ^18^ | |
| Additional risk ratio per decade of life | 1.97 | 1.79 | 2.16 | Triangular | ^69^ | |
| **Risk of CTEPH** |  |  |  |  |  | |
| Cumulative rate, 5 years | 2.50% | 2.00% | 3.30% | Beta | ^32^ | |
| **Hazard ratio for excess mortality compared to general population** |  |  |  |  |  | |
| Index VTE* | 4.41 | 3.63 | 5.36 | Triangular | ^18^ | |
| IC bleed* | 1.30 | 0.98 | 1.73 | Triangular | ^18^ | |
| CTEPH* | 2.60 | 2.20 | 5.60 | Triangular | ^18^ | |
| The listed clinical outcome probabilities were applied for both groups assuming treatment with anticoagulation. *Excluding events modelled. VTE = venous thromboembolism; AC = anticoagulation; PE = pulmonary embolism; DVT = deep vein thrombosis; IC = intracranial; EC = extracranial; CRNM = clinically relevant non-major; CTEPH = chronic thromboembolic pulmonary hypertension. **Supplemental Table 9.** Utilities and disutilities for all care phases \| \| \| \| \| \|; **Variable** \| **Duration** \| **Base** \| **Low** \| **High** \| **Distribution** \| **Source**; **Applied in PE management phase** \| \| \| \| \| \|; **Utilities** \| \| \| \| \| \|; Baseline \| Variable \| 0.851 \| 0.839 \| 0.863 \| Beta \| ^75^; **Disutilities** \| \| \| \| \| \|; CD, Cardiac arrest \| 30 days \| 0.402 \| 0.362 \| 0.442 \| Beta \| ^76-79,^*; CD, High grade AV block \| 14 days \| 0.124 \| 0.112 \| 0.137 \| Beta \| ^80, 81,^*; CD, Respiratory failure \| 10 days \| 0.500 \| 0.450 \| 0.550 \| Beta \| ^59, 82,^*; CD, Increased oxygen needs \| 5 days \| 0.400 \| 0.360 \| 0.440 \| Beta \| ^59,^*; CD, Hypotension \| 5 days \| 0.025 \| 0.023 \| 0.028 \| Beta \| ^83,^*; BO to any treatment \| 5 days \| 0.150 \| 0.100 \| 0.200 \| Beta \| ^84,†^; AC treatment, apixaban \| Variable \| 0.002 \| 0.000 \| 0.006 \| Beta \| ^18, 27^; Readmission \| 5 days \| 0.100 \| 0.090 \| 0.110 \| Beta \| ^85-87,^*; ICU stay \| Variable \| 0.305 \| 0.275 \| 0.336 \| Beta \| ^3, 88,^*; Non-ICU stay \| Variable \| 0.125 \| 0.113 \| 0.138 \| Beta \| ^3, 88,^*; **Applied in long-term care phase** \| \| \| \| \| \|; **Utilities** \| \| \| \| \| \|; CTEPH \| Variable \| 0.560 \| 0.528 \| 0.592 \| Beta \| ^89-92^; **Disutilities** \| \| \| \| \| \|; Recurrent PE \| 30 days \| 0.3200 \| 0.0900 \| 0.5900 \| Beta \| ^18, 27, 93, 94^; Recurrent DVT \| 30 days \| 0.1100 \| 0.0000 \| 0.3100 \| Beta \| ^18, 27, 93, 94^; **Applied during either care phase** \| \| \| \| \| \|; **Utilities** \| \| \| \| \| \|; Death \| Not applicable \| 0.000 \| 0.000 \| 0.000 \| Beta \| ^95^; **Disutilities** \| \| \| \| \| \|; Major EC bleed \| 30 days \| 0.3000 \| 0.0900 \| 0.4600 \| Beta \| ^18, 27, 93^; Major IC bleed \| 30 days \| 0.6000 \| 0.5400 \| 0.6600 \| Beta \| ^18, 96,^*; CRNM bleed \| 2 days \| 0.0054 \| 0.0000 \| 0.0195 \| Beta \| ^18, 27, 97, 98^; *Lower and upper bounds estimated as ±10% of base value. †Lower and upper bounds estimated as ±50% of base value. PE = pulmonary embolism; CD = clinical deterioration; BO = bailout; AC = anticoagulation; ICU = intensive care unit; CTEPH = chronic thromboembolic pulmonary hypertension; DVT = deep vein thrombosis; EC = extracranial; IC = intracranial; CRNM = clinically relevant non-major. \| \| \| \| \| \| | | | | | | |

| **Supplemental Table 10.** Costs associated with PE management phase health states | | | | | |
| --- | --- | --- | --- | --- | --- |
| **Variable** | **Base** | **Low** | **High** | **Distribution** | **Source** |
| **All-cause mortality*** | $0 | - | - | Fixed | Assumption |
| **Major bleed*** |  |  |  |  |  |
| IC bleed, nonfatal | $27,825.93 | $25,043.34 | $30,608.52 | Lognormal | ^45-47,†^ |
| EC bleed, nonfatal | $21,430.00 | $19,287.00 | $23,573.00 | Lognormal | ^7, 12, 45, 48,†^ |
| **CDBO*** |  |  |  |  |  |
| CD, cardiac arrest | $21,650.69 | $19,485.62 | $23,815.76 | Lognormal | ^35,†^ |
| CD, high grade AV block | $16,913.94 | $15,222.55 | $18,605.33 | Lognormal | ^49, 102,†^ |
| CD, respiratory failure | $3,990.42 | $3,591.38 | $4,389.46 | Lognormal | ^35,†^ |
| CD, increased oxygen needs | $1,925.51 | $1,732.96 | $2,118.06 | Lognormal | ^50,†^ |
| CD, hypotension | $4,960.47 | $4,464.42 | $5,456.52 | Lognormal | ^35,†^ |
| BO, LBMT | $17,624.70 | $15,862.23 | $19,387.17 | Lognormal | ^3, 13,†^ |
| BO, systemic thrombolysis | $14,235.57 | $12,812.01 | $15,659.12 | Lognormal | ^3, 13, 51,†^ |
| **CRNM bleed*** | $5,371.19 | $4,834.07 | $5,908.31 | Lognormal | ^7, 45,†^ |
| **No complications*** | $0 | - | - | Fixed | Assumption |
| ^*^Measured at the sooner of hospital discharge or 7 days following the procedure. ^†^Lower and upper bounds estimated as ±10% of base value. PE = pulmonary embolism; IC = intracranial; EC = extracranial; CDBO = clinical deterioration and/or bailout; CD = clinical deterioration; AV = atrioventricular; BO = bailout; LBMT = large bore mechanical thrombectomy; CRNM = clinically relevant non-major. | | | | | |

| **Supplemental Table 11.** Microcosting of treatment in PE management phase | | | | | |
| --- | --- | --- | --- | --- | --- |
| **Variable** | **Base** | **Low** | **High** | **Distribution** | **Source** |
|  | **Procedure, LBMT** | | | | |
| Catheterization lab, per procedure | $4,717.32 | $4,245.59 | $5,189.05 | Lognormal | ^3, 12,^* |
| Device, LBMT catheter | $11,900.77 | $10,710.69 | $13,090.85 | Lognormal | ^11, 12,^* |
| Introducer sheath, Gore DrySeal | $372.00 | $334.80 | $409.20 | Lognormal |  |
| Catheter, Berman for PA gram | $110.00 | $99.00 | $121.00 | Lognormal | ^13,^* |
| Guide wire, Amplatz Super Stiff | $35.00 | $31.50 | $38.50 | Lognormal | ^13,^* |
| Vascular dilator kit, Sorin | $165.00 | $148.50 | $181.50 | Lognormal | ^13,^* |
| Vascular closure, Perclose | $201.00 | $180.90 | $221.10 | Lognormal | ^13,^* |
| Misc, mattress suture + red rubber | $6.64 | $5.98 | $7.30 | Lognormal | ^13,^* |
|  | **Procedure, CDT** | | | | |
| Catheterization lab, per procedure | $3,305.16 | $2,974.64 | $3,635.68 | Lognormal | ^3, 12,^* |
| Device, US-CDT catheter (x2) | $5,964.53 | $2,595.00 | $3,109.47 | Lognormal | ^11-13, 35^ |
| Device, side-hole CDT catheter | $112.80 | $71.90 | $140.05 | Lognormal | ^11, 13, 35^ |
| Device, side-slit CDT catheter | $134.80 | $115.32 | $154.25 | Lognormal | ^35, 52^ |
| Device, pharmacomechanical CDT catheter | $2,486.70 | $1,357.91 | $3,442.88 | Lognormal | ^11, 35^ |
| Device, gradient side-hole CDT catheter | $136.30 | $122.94 | $150.26 | Lognormal | ^35, 53^ |
| tPA, 10mg vial | $1,000.13 | $900.12 | $1,141.60 | Lognormal | ^13, 51,^* |
| tPA, 50mg vial | $4,067.53 | $3,660.78 | $4,642.89 | Lognormal | ^13, 51,^* |
| Guidewire, J-tip 240 | $17.55 | $15.80 | $19.31 | Lognormal |  |
| Misc, next day x-ray imaging | $99.84 | $89.86 | $109.82 | Lognormal | ^13,^* |
| Misc, fibrinogen labs (every 8 hours) | $36.99 | $33.29 | $40.69 | Lognormal | ^13,^* |
| Misc, blood gas labs (every 8 hours) | $96.24 | $86.62 | $105.86 | Lognormal | ^13,^* |
|  | **Procedure, both** | | | | |
| Catheterization lab, per minute | $50.62 | $45.55 | $55.68 | Lognormal | ^12, 54, 55,^* |
| Introducer sheath, 7 Fr | $9.25 | $8.33 | $10.18 | Lognormal | ^13,^* |
| Catheter, Swan Ganz | $38.00 | $34.20 | $41.80 | Lognormal | ^13,^* |
| Catheter, Pigtail for PA gram | $8.00 | $7.20 | $8.80 | Lognormal | ^13,^* |
| Misc, micropuncture access kit | $17.95 | $16.16 | $19.75 | Lognormal |  |
| Misc, catheterization sterile packet | $20.00 | $18.00 | $22.00 | Lognormal | ^13,^* |
| Misc, ultrasound probe cover | $9.50 | $8.55 | $10.45 | Lognormal | ^13,^* |
| Misc, contrast dye | $14.00 | $12.60 | $15.40 | Lognormal | ^13,^* |
| Misc, procedural medications | $3.76 | $3.38 | $4.14 | Lognormal | ^13,^* |
|  | **Peri-procedure, both** | | | | |
| Stay, inpatient, per day | $2,771.71 | $1,819.07 | $3,151.00 | Lognormal | ^12, 56-59^ |
| Stay, ICU, per day | $5,867.44 | $2,617.76 | $8,760.00 | Lognormal | ^12, 59-61^ |
| Stay, readmission, per event | $24,267.70 | $17,662.00 | $26,469.00 | Lognormal | ^12, 62-65^ |
| Misc, daily lab tests | $139.44 | $125.50 | $153.38 | Lognormal | ^13,^* |
| Misc, echocardiogram | $464.00 | $210.00 | $1,064.00 | Lognormal | ^13, 66^ |
| Misc, heparin infusion on floor per day | $7.72 | $6.95 | $8.49 | Lognormal | ^13,^* |
| Misc, AC induction period, per day | $37.77 | $33.99 | $41.55 | Lognormal | ^67, 68,^* |
| Misc, AC long-term maintenance, per day | $18.89 | $17.00 | $20.77 | Lognormal | ^67, 68,^* |
| *Lower and upper bounds estimated as ±10% of base value. PE = pulmonary embolism; LBMT = large bore mechanical thrombectomy; PA = pulmonary angiogram; CDT = catheter directed thrombolysis; US-CDT = ultrasound facilitated catheter directed thrombolysis; ICU = intensive care unit; AC = anticoagulation. | | | | | |

| **Supplemental Table 12.** Resource utilization from PE management phase | | | | | | |
| --- | --- | --- | --- | --- | --- | --- |
| **Variable** | **Time** | **Base** | **Low** | **High** | **Distribution** | **Source** |
|  | **LBMT** | | | | | |
| Procedure time, minutes | a | 93.2 | 88.9 | 97.5 | Triangular | ^3^ |
| Post-procedure ICU LOS, days^†^ | b | 0.61 | 0.48 | 0.74 | Triangular | ^3^ |
| Total hospital LOS, days^†^ | b | 4.50 | 4.15 | 4.82 | Triangular | ^3^ |
| All-cause mortality | c | 0.4% | 0.0% | 2.2% | Beta | ^3^ |
| All-cause readmission | c | 3.2% | 1.4% | 6.2% | Beta | ^3^ |
|  | **CDT** | | | | | |
| Proportion with bilateral CDT | a | 92.0% | 88.2% | 94.9% | Beta | ^3^ |
| Proportion receiving tPA | a | 99.3% | 97.4% | 99.9% | Beta | ^3^ |
| tPA dose, mg | a | 18.90 | 17.81 | 20.01 | Triangular | ^3^ |
| Procedure time, minutes | a | 65.3 | 60.2 | 70.3 | Triangular | ^3^ |
| Post-procedure ICU LOS, days^†^ | b | 1.71 | 1.57 | 1.85 | Triangular | ^3^ |
| Total hospital LOS, days^†^ | b | 5.30 | 4.84 | 5.76 | Triangular | ^3^ |
| All-cause mortality | c | 0.8% | 0.1% | 3.0% | Beta | ^3^ |
| All-cause readmission | c | 7.9% | 4.9% | 12.1% | Beta | ^3^ |
| ^a^Measured at the sooner of hospital discharge or 7 days following the procedure. ^b^Measured in the hospitalization associated with the PE procedure. ^c^Measured within 30-days of the PE procedure. *Lower and upper bounds estimated as ±10% of base value. †Mean number of days that included overnight stay. PE = pulmonary embolism; LBMT = large bore mechanical thrombectomy; ICU = intensive care unit; LOS = length of stay; CDT = catheter directed thrombolysis; tPA = tissue plasminogen activator. | | | | | | |

| **Supplemental Table 13.** Costs associated with long-term care phase health states | | | | | |
| --- | --- | --- | --- | --- | --- |
| **Variable** | **Base** | **Low** | **High** | **Distribution** | **Source** |
| **Major bleed** |  |  |  |  |  |
| IC bleed, nonfatal | $27,825.93 | $25,043.34 | $30,608.52 | Lognormal | ^7, 45, 47,^* |
| Post-IC bleed, rehabilitation (1 cycle) | $18,363.39 | $16,527.05 | $20,199.73 | Lognormal | ^18, 70,^* |
| Post-IC bleed, long-term care (per cycle) | $5,693.91 | $5,124.52 | $6,263.30 | Lognormal | ^18, 21, 71,^* |
| EC bleed, nonfatal | $21,430.00 | $19,287.00 | $23,573.00 | Lognormal | ^7, 12, 45, 48,^* |
| Mortality, fatal bleed | $27,825.93 | $25,043.34 | $30,608.52 | Lognormal | ^7, 27, 45, 47,^* |
| **CRNM bleed** | $5,371.19 | $4,834.07 | $5,908.31 | Lognormal | ^7, 45,^* |
| **Recurrent VTE** |  |  |  |  |  |
| Recurrent PE | $20,608.84 | $18,547.96 | $22,669.72 | Lognormal | ^7, 45,^* |
| Recurrent DVT | $9,781.91 | $8,803.72 | $10,760.10 | Lognormal | ^7, 45,^* |
| Mortality, VTE-related | $20,608.84 | $18,547.96 | $22,669.72 | Lognormal | ^7, 27, 45,^* |
| **CTEPH** |  |  |  |  |  |
| Initial diagnosis and care | $7,708.49 | $6,937.64 | $8,479.34 | Lognormal | ^7, 72,^* |
| Post-CTEPH, long-term care (per cycle) | $10,370.01 | $9,333.01 | $11,407.01 | Lognormal | ^18, 21, 73, 74,^* |
| *Lower and upper bounds estimated as ±10% of base value. IC = intracranial; EC = intracranial; CRNM = clinically relevant non-major; PE = pulmonary embolism; DVT = deep vein thrombosis; VTE = venous thromboembolism; CTEPH = chronic thromboembolic pulmonary hypertension. | | | | | |

**Supplemental Methods: Systematic literature review of device-specific CTEPH rates**

**Overview**

Data pertaining to CTEPH incidence following each treatment were derived from published clinical literature. The literature search follows the Preferred Reporting Items for Systematic Reviews and Meta-Analyses (PRISMA) methodology and was conducted using the PubMed database^99^. The search term strategy is described below. Records were screened and excluded based on the appropriateness of their title or abstract, and full-text references were subsequently evaluated for suitability. Inclusion criteria included studies of intermediate- and high-risk PE treated with either LBMT or CDT that reported CTEPH outcomes and were available in English. Exclusion criteria included editorials, review articles, case reports or case series with fewer than 10 patients, interim and subanalyses, and research involving pediatric patients. Studies were not required to have a prespecified length of follow-up for inclusion in the systematic review. However, the reported follow-up had to exceed one year for a study’s data to be used in the long-term model.

**Search structure**

| Database | PubMed |
| --- | --- |
| Timeframe | All dates through January 22, 2025 |
| Objective | Identifying CTEPH outcomes for large-bore mechanical thrombectomy and catheter-directed thrombolytics in the treatment of intermediate- and high-risk PE |

|  | ( |
| --- | --- |
| (a) | (“Pulmonary Embolism”[MeSH Terms]) OR (Pulmonary Embolism[Text Word]) |
|  | ) AND ( ( |
| (b) | (Thrombectomy[MeSH Terms]) OR (mechanical thrombectomy[Text Word]) OR (large bore aspiration thrombectomy[Text Word]) OR (endovascular thrombectomy[Text Word]) |
|  | ) OR ( |
| (c) | (catheter directed thrombolysis[Text Word]) OR (catheter directed thrombolytics[Text Word]) OR (catheter based thrombolysis[Text Word]) OR (percutaneous thrombolysis[Text Word]) OR (ultrasound assisted thrombolysis[Text Word]) OR (ultrasound assisted catheter directed thrombolysis[Text Word]) OR (Catheter-directed Ultrasound Assisted Thrombolysis[Text Word]) OR (USAT[Text Word]) OR (Ultrasound-assisted catheter-directed thrombolysis[Text Word]) OR (Ultrasound-Assisted[Text Word]) OR (Ultrasound-facilitated[Text Word]) OR (USCDT[Text Word]) OR (EKOS[Text Word]) OR (EKOSONIC[Text Word]) |
|  | ) ) AND ( |
| (d) | (“Hypertension, Pulmonary”[MeSH Terms]) OR (Chronic Thromboembolic Pulmonary Hypertension[Text Word]) OR (Pulmonary Hypertension[Text Word]) OR (CTEPH[Text Word]) OR (CTPH[Text Word]) |
|  | ) |

**PRISMA diagram and model inclusion**

Records identified through PubMed search (n=134)

Records screened (n=134)

Records removed (n=121):

- No mention of relevant interventions (n=84)
- No focus on CTEPH outcomes (n=35)

Full-text articles assessed for eligibility (n=15)

Articles included for consideration (n=4)

Full-text articles excluded (n=11):

- Narrative review (n=2)
- Case-series (n=3)
- No CTEPH outcomes (n=4)
- No results by device type (n=1)
- Substantial missing data (n=1)

Articles included in the model (n=1)

Articles excluded from the model (n=3):

- No long-term follow-up (>1 year) (n=1)
- No long-term follow-up (>1 year) and unclear CTEPH definition (n=2)

**Search results**

| **Source** | **Design** | **N** | **Outcome** | **Inclusion**  **In Model** | **Notes** |
| --- | --- | --- | --- | --- | --- |
| ^5^ | Prospective, single-arm, multi-center | LBMT = 800 | CTEPH at 6 months:  6/581 (1.03%) | N | Excluded from model because follow-up did not exceed 1 year |
| ^33^ | Retrospective, multi-center, EHR, propensity score matched | MT = 1,102   CDT = 1,102 | MT CTEPH at 5 years:  20/1102 (1.8%)  CDT CTEPH at 5 years:  38/1102 (3.4%) | Y | Included in scenario analysis due to 5-year follow-up and propensity score matching |
| ^100^ | Retrospective, single-center | CDT = 283 | Chronic PH at 1 year:  14/282 (6.3%) | N | Excluded from model because follow-up did not exceed 1 year and unclear CTEPH definition |
| ^101^ | Retrospective, single-arm, multi-center | US-CDT = 102 | Chronic PH at 6 months:  1/70 (1.43%) | N | Excluded from model because follow-up did not exceed 1 year and unclear CTEPH definition |
| LBMT = large bore mechanical thrombectomy; CTEPH = chronic thromboembolic pulmonary hypertension; MT = mechanical thrombectomy; CDT = catheter directed thrombolysis; US-CDT = ultrasound facilitated catheter directed thrombolysis. | | | | | |

**Supplemental Methods: Model validation**

Model validation was conducted following ISPOR-SMDM good practices recommendations for health economic modeling.^38^ Four types of validations were performed: 1) face validity, 2) verification (or internal validity), 3) cross validity, 4) external validity. Predictive validation was not feasible due to the absence of prospective long-term data.

**Face validity**

Face validity was assessed by clinical experts in PE intervention and health economists. Reviewing authors examined the combined model approach, the selection of health states, and key assumptions (e.g., cost estimates, long-term event rates, and scenario analyses). The model structure was agreed to accurately represent current clinical practices and economic evaluation standards. The best available data sources were implemented when available, including the application of PEERLESS RCT data for all acute phase outcomes, extrapolated RCT evidence for long-term outcomes, and a systematic literature review for the scenario analysis evaluating differential CTEPH incidence. The resource-based accounting approach was based on established frameworks in published literature. The representation of continuous disease progression as discrete health states with fixed transition intervals was identified as a simplification of clinical reality but is a standard implementation practice in state-transition models. Reviewing author feedback was implemented in the model to ensure its accuracy in reflecting clinical practice.

**Verification (internal validity)**

The model was verified through a systematic review of all calculations and formulas. Input parameters were checked against their sources. A health economist author reviewed the implementation and coding in Excel and compared selected outputs with hand calculations. Individual simulated patients were traced through the model structure to confirm that health state transitions were correctly implemented. All model parameters were tested in sensitivity analysis and at extreme values to ensure that model outputs changed in the anticipated direction and by reasonable magnitudes.

**Cross validation**

Findings were compared to other published cost analyses of reperfusion strategies for pulmonary embolism, including studies evaluating the costs of CDT and mechanical thrombectomy. While absolute cost estimates differed across studies due to variation in resource cost and utilization assumptions, the direction and magnitude of prior findings were consistent with the present analysis^11-13^. Additionally, the model structure applied in this study was based on established VTE modeling frameworks and parameterization was consistent with published literature.

**External validation**

Model outcomes from the PE treatment phase were validated against the clinical outcomes reported in the PEERLESS RCT; specifically evaluating against trial-reported clinical event rates (e.g., mortality, ICU length of stay, 30-day readmission). Model-predicted outcomes fell within the expected ranges of trial results (i.e., within the 95% confidence intervals), confirming the model’s accuracy in reproducing real-world clinical endpoints.

# **References**

1.	Konstantinides SV, Meyer G, Becattini C, Bueno H, Geersing G-J, Harjola V-P, Huisman MV, Humbert M, Jennings CS and Jiménez D. 2019 ESC Guidelines for the diagnosis and management of acute pulmonary embolism developed in collaboration with the European Respiratory Society (ERS) The Task Force for the diagnosis and management of acute pulmonary embolism of the European Society of Cardiology (ESC). *European heart journal*. 2020;41:543-603.

2.	Giri J, Sista AK, Weinberg I, Kearon C, Kumbhani DJ, Desai ND, Piazza G, Gladwin MT, Chatterjee S and Kobayashi T. Interventional therapies for acute pulmonary embolism: current status and principles for the development of novel evidence: a scientific statement from the American Heart Association. *Circulation*. 2019;140:e774-e801.

3.	Jaber WA, Gonsalves CF, Stortecky S, Horr S, Pappas O, Gandhi RT, Pereira K, Giri J, Khandhar SJ, Ammar KA, Lasorda DM, Stegman B, Busch L, Dexter II DJ, Azene EM, Daga N, Elmasri F, Kunavarapu CR, Rea ME, Rossi JS, Campbell J, Lindquist J, Raskin A, Smith JC, Tamlyn TM, Hernandez GA, Rali P, Schmidt TR, Bruckel JT, Camacho JC, Li J, Selim S, Toma C, Basra SS, Bergmark BA, Khalsa B, Zlotnick DM, Castle J, O'Connor DJ and Gibson CM. Large-bore Mechanical Thrombectomy Versus Catheter-directed Thrombolysis in the Management of Intermediate-risk Pulmonary Embolism: Primary Results of the PEERLESS Randomized Controlled Trial. *Circulation*. 2025;151:260-273.

4.	Toma C, Jaber WA, Weinberg MD, Bunte MC, Khandhar S, Stegman B, Gondi S, Chambers J, Amin R and Leung DA. Acute outcomes for the full US cohort of the FLASH mechanical thrombectomy registry in pulmonary embolism. *EuroIntervention*. 2023;18:1201.

5.	Khandhar S, Jaber W, Bunte MC, Cho K, Weinberg MD, Mina B, Stegman B, Pollak J, Khosla A and Elmasri F. Longer-term outcomes following mechanical thrombectomy for intermediate-and high-risk pulmonary embolism: 6-month FLASH registry results. *Journal of the Society for Cardiovascular Angiography & Interventions*. 2023;2:101000.

6.	Chatterjee S, Chakraborty A, Weinberg I, Kadakia M, Wilensky RL, Sardar P, Kumbhani DJ, Mukherjee D, Jaff MR and Giri J. Thrombolysis for pulmonary embolism and risk of all-cause mortality, major bleeding, and intracranial hemorrhage: a meta-analysis. *Jama*. 2014;311:2414-2421.

7.	Geller BJ, Adusumalli S, Pugliese SC, Khatana SAM, Nathan A, Weinberg I, Jaff MR, Kobayashi T, Mazurek JA and Khandhar S. Outcomes of catheter-directed versus systemic thrombolysis for the treatment of pulmonary embolism: a real-world analysis of national administrative claims. *Vascular Medicine*. 2020;25:334-340.

8.	Tapson VF, Sterling K, Jones N, Elder M, Tripathy U, Brower J, Maholic RL, Ross CB, Natarajan K and Fong P. A randomized trial of the optimum duration of acoustic pulse thrombolysis procedure in acute intermediate-risk pulmonary embolism: the OPTALYSE PE trial. *JACC: Cardiovascular Interventions*. 2018;11:1401-1410.

9.	Piazza G, Hohlfelder B, Jaff MR, Ouriel K, Engelhardt TC, Sterling KM, Jones NJ, Gurley JC, Bhatheja R and Kennedy RJ. A prospective, single-arm, multicenter trial of ultrasound-facilitated, catheter-directed, low-dose fibrinolysis for acute massive and submassive pulmonary embolism: the SEATTLE II study. *Cardiovascular Interventions*. 2015;8:1382-1392.

10.	Kazi DS, Abdullah AR, Arnold SV, Basu A, Bellows BK, Breathett K, Chew DS, Cohen DJ, DeJong C and Heidenreich PA. 2025 AHA/ACC Statement on Cost/Value Methodology in Clinical Practice Guidelines (Update From 2014 Statement): A Report of the American College of Cardiology/American Heart Association Joint Committee on Clinical Practice Guidelines. *JACC*. 2025.

11.	Callese T, Moriarty J, Maehara C, Cusumano L, Mathevosian S, Enzmann D, Padia S and Srinivasa R. Cost drivers in endovascular pulmonary embolism interventions. *Clinical Radiology*. 2023;78:e143-e149.

12.	Noman A, Stegman B, DuCoffe AR, Bhat A, Hoban K and Bunte MC. Episode Care Costs Following Catheter-Directed Reperfusion Therapies for Pulmonary Embolism: A Literature-Based Comparative Cohort Analysis. *The American Journal of Cardiology*. 2024.

13.	Tran A, Toma C, Jaber W, Heintz J, Matthai WH, Palevsky H, Giri J, Kobayashi TJ, Nathan AS and Baumgartner S. Cost-Comparison of Mechanical Thrombectomy and Catheter-Directed Thrombolysis in Intermediate-Risk Pulmonary Embolism. *Journal of the Society for Cardiovascular Angiography & Interventions*. 2024;3:101187.

14.	Husereau D, Drummond M, Augustovski F, de Bekker-Grob E, Briggs AH, Carswell C, Caulley L, Chaiyakunapruk N, Greenberg D and Loder E. Consolidated Health Economic Evaluation Reporting Standards 2022 (CHEERS 2022) statement: updated reporting guidance for health economic evaluations. *International journal of technology assessment in health care*. 2022;38:e13.

15.	Kim DD, Do LA, Synnott PG, Lavelle TA, Prosser LA, Wong JB and Neumann PJ. Developing criteria for health economic quality evaluation tool. *Value in Health*. 2023;26:1225-1234.

16.	Sanders GD, Neumann PJ, Basu A, Brock DW, Feeny D, Krahn M, Kuntz KM, Meltzer DO, Owens DK and Prosser LA. Recommendations for conduct, methodological practices, and reporting of cost-effectiveness analyses: second panel on cost-effectiveness in health and medicine. *Jama*. 2016;316:1093-1103.

17.	Lanitis T, Leipold R, Hamilton M, Rublee D, Quon P, Browne C and Cohen AT. Cost-effectiveness of apixaban versus low molecular weight heparin/vitamin k antagonist for the treatment of venous thromboembolism and the prevention of recurrences. *BMC Health Services Research*. 2017;17:1-14.

18.	Lanitis T, Leipold R, Hamilton M, Rublee D, Quon P, Browne C and Cohen AT. Cost-effectiveness of apixaban versus other oral anticoagulants for the initial treatment of venous thromboembolism and prevention of recurrence. *Clinical therapeutics*. 2016;38:478-493. e16.

19.	de Jong LA, Dvortsin E, Janssen KJ and Postma MJ. Cost-effectiveness analysis for apixaban in the acute treatment and prevention of venous thromboembolism in the Netherlands. *Clinical therapeutics*. 2017;39:288-302. e4.

20.	Folkerts K, Broughton J, Sheikh U and Mckaig S. Cost-effectiveness of rivaroxaban versus apixaban for the initial treatment of venous thromboembolism and extended prevention of recurrences in the UK. *Journal of Medical Economics*. 2019;22:1179-1191.

21.	Mason SE, Zhu J, Rahaghi FN, Washko GR and Pandya A. Cost-effectiveness microsimulation of catheter-directed thrombolysis in submassive pulmonary embolism using a right ventricular function model. *Journal of thrombosis and thrombolysis*. 2020;49:673-680.

22.	Kabrhel C, Ali A, Choi JG and Hur C. Systemic Thrombolysis, Catheter‐Directed Thrombolysis, and Anticoagulation for Intermediate‐risk Pulmonary Embolism: A Simulation Modeling Analysis. *Academic Emergency Medicine*. 2017;24:1235-1243.

23.	Perlroth DJ, Sanders GD and Gould MK. Effectiveness and cost-effectiveness of thrombolysis in submassive pulmonary embolism. *Archives of internal medicine*. 2007;167:74-80.

24.	Stevens SM, Woller SC, Kreuziger LB, Bounameaux H, Doerschug K, Geersing G-J, Huisman MV, Kearon C, King CS and Knighton AJ. Antithrombotic therapy for VTE disease: second update of the CHEST guideline and expert panel report. *Chest*. 2021;160:e545-e608.

25.	Gonsalves CF, Gibson CM, Stortecky S, Alvarez RA, Beam DM, Horowitz JM, Silver MJ, Toma C, Rundback JH and Rosenberg SP. Randomized controlled trial of mechanical thrombectomy vs catheter-directed thrombolysis for acute hemodynamically stable pulmonary embolism: Rationale and design of the PEERLESS study. *American Heart Journal*. 2023;266:128-137.

26.	Kaatz S, Ahmad D, Spyropoulos A and Schulman S. Definition of clinically relevant non‐major bleeding in studies of anticoagulants in atrial fibrillation and venous thromboembolic disease in non‐surgical patients: communication from the SSC of the ISTH. *Journal of Thrombosis and Haemostasis*. 2015;13:2119-2126.

27.	National Institute for Health and Care Excellence (NICE). Apixaban for the treatment and secondary prevention of deep vein thrombosis and/or pulmonary embolism [Technology appraisal guidance: TA341]. 2015.

28.	Zhang RS, Zhang P, Yuriditsky E, Taslakian B, Rhee AJ, Greco AA, Elbaum L, Mukherjee V, Postelnicu R and Amoroso NE. Comparing Real‐World Outcomes of Catheter‐Directed Thrombolysis and Catheter‐Based Thrombectomy in Acute Pulmonary Embolism: A Post PEERLESS Analysis. *Catheterization and Cardiovascular Interventions*. 2024.

29.	Agnelli G, Buller HR, Cohen A, Curto M, Gallus AS, Johnson M, Masiukiewicz U, Pak R, Thompson J and Raskob GE. Oral apixaban for the treatment of acute venous thromboembolism. *New England Journal of Medicine*. 2013;369:799-808.

30.	Agnelli G, Buller HR, Cohen A, Curto M, Gallus AS, Johnson M, Porcari A, Raskob GE and Weitz JI. Apixaban for extended treatment of venous thromboembolism. *New England Journal of Medicine*. 2013;368:699-708.

31.	Prandoni P, Noventa F, Ghirarduzzi A, Pengo V, Bernardi E, Pesavento R, Iotti M, Tormene D, Simioni P and Pagnan A. The risk of recurrent venous thromboembolism after discontinuing anticoagulation in patients with acute proximal deep vein thrombosis or pulmonary embolism. A prospective cohort study in 1,626 patients. *Haematologica*. 2007;92:199-205.

32.	Luijten D, Talerico R, Barco S, Cannegieter SC, Delcroix M, Ende-Verhaar YM, Huisman MV, Konstantinidis S, Mairuhu ATA, van Mens TE, Ninaber M, Pruszczyk P, Vonk Noordegraaf A and Klok FA. Incidence of chronic thromboembolic pulmonary hypertension after acute pulmonary embolism: an updated systematic review and meta-analysis. *European Respiratory Journal*. 2023;62:2300449.

33.	Tsukagoshi J, Wick B, Karim A, Khanipov K and Cox MW. Perioperative and intermediate outcomes of patients with pulmonary embolism undergoing catheter-directed thrombolysis vs percutaneous mechanical thrombectomy. *Journal of Vascular Surgery: Venous and Lymphatic Disorders*. 2024;12:101958.

34.	Arias E, Xu J and Kochanek K. United States life tables, 2021. 2023.

35.	PINC AI™ Applied Sciences Premier Inc. PINC AI™ Healthcare Database: Data that informs and performs (White Paper). September 2024. 2024.

36.	U.S. Bureau of Labor Statistics data on Consumer Price Index (CPI) for medical care (calculator on Official Data Foundation / Alioth LLC platform). 2024.

37.	U.S. Department of Veterans Affairs Health Economics Resource Center. Measuring Costs for Cost-Effectiveness Analysis.

38.	Eddy DM, Hollingworth W, Caro JJ, Tsevat J, McDonald KM and Wong JB. Model transparency and validation: a report of the ISPOR-SMDM Modeling Good Research Practices Task Force–7. *Medical Decision Making*. 2012;32:733-743.

39.	Silver MJ, Gibson CM, Giri J, Khandhar S, Jaber W, Toma C, Mina B, Bowers T, Greenspon L and Kado H. Outcomes in high-risk pulmonary embolism patients undergoing FlowTriever mechanical thrombectomy or other contemporary therapies: results from the FLAME study. *Circulation: Cardiovascular Interventions*. 2023;16:e013406.

40.	Zhang RS, Keller N, Yuriditsky E, Bailey E, Elbaum L, Leiva O, Greco AA, Postelnicu R, Li V and Hena KM. Mitigating health disparities by improving access to catheter-based therapies for vulnerable patients with acute pulmonary embolism. *Cardiovascular Revascularization Medicine*. 2024.

41.	Magnuson EA, Chinnakondepalli K, Vilain K, Kearon C, Julian JA, Kahn SR, Goldhaber SZ, Jaff MR, Kindzelski AL and Herman K. Cost-effectiveness of pharmacomechanical catheter-directed thrombolysis versus standard anticoagulation in patients with proximal deep vein thrombosis: results from the ATTRACT trial. *Circulation: Cardiovascular Quality and Outcomes*. 2019;12:e005659.

42.	Reynolds MR, Magnuson EA, Wang K, Lei Y, Vilain K, Walczak J, Kodali SK, Lasala JM, O'Neill WW and Davidson CJ. Cost-effectiveness of transcatheter aortic valve replacement compared with standard care among inoperable patients with severe aortic stenosis: results from the placement of aortic transcatheter valves (PARTNER) trial (Cohort B). *Circulation*. 2012;125:1102-1109.

43.	Cohen DJ, Krumholz HM, Sukin CA, Ho KK, Siegrist RB, Cleman M, Heuser RR, Brinker JA, Moses JW and Savage MP. In-hospital and one-year economic outcomes after coronary stenting or balloon angioplasty: results from a randomized clinical trial. *Circulation*. 1995;92:2480-2487.

44.	Cohen DJ, Bakhai A, Shi C, Githiora L, Lavelle T, Berezin RH, Leon MB, Moses JW, Carrozza Jr JP and Zidar JP. Cost-effectiveness of sirolimus-eluting stents for treatment of complex coronary stenoses: results from the Sirolimus-Eluting Balloon Expandable Stent in the Treatment of Patients With De Novo Native Coronary Artery Lesions (SIRIUS) trial. *Circulation*. 2004;110:508-514.

45.	Preblick R, Kwong WJ, White RH and Goldhaber SZ. Cost-effectiveness of edoxaban for the treatment of venous thromboembolism based on the Hokusai-VTE study. *Hospital practice*. 2015;43:249-257.

46.	Li A, Carlson JJ, Kuderer NM, Schaefer JK, Li S, Garcia DA, Khorana AA, Carrier M and Lyman GH. Cost‐effectiveness analysis of low‐dose direct oral anticoagulant (DOAC) for the prevention of cancer‐associated thrombosis in the United States. *Cancer*. 2020;126:1736-1748.

47.	Tong X, George MG, Gillespie C and Merritt R. Trends in hospitalizations and cost associated with stroke by age, United States 2003–2012. *International journal of stroke*. 2016;11:874-881.

48.	Wells PS, Prins MH, Beyer-Westendorf J, Lensing AW, Haskell L, Levitan B, Laliberté F, Ashton V, Xiao Y and Lejeune D. Health-care cost impact of continued anticoagulation with rivaroxaban vs aspirin for prevention of recurrent symptomatic VTE in the EINSTEIN-CHOICE Trial Population. *Chest*. 2018;154:1371-1378.

49.	Beller JP, Tyerman Z, Mehaffey JH, Hawkins RB, Charles EJ, Yarboro LT, Teman NR, Wancheck T, Ailawadi G and Mehta NK. Early versus delayed pacemaker for heart block after valve surgery: a cost-effectiveness analysis. *Journal of Surgical Research*. 2021;259:154-162.

50.	Kelton K, Klein T, Murphy D, Belger M, Hille E, McCollam PL, Spiro T and Burge R. Cost-effectiveness of combination of baricitinib and remdesivir in hospitalized patients with COVID-19 in the United States: a modelling study. *Advances in Therapy*. 2022;39:562-582.

51.	Drugs.com. Activase (alteplase) prices, $4,642.89 for 50mg vial, March 2023. 2023.

52.	DOTmed.com Inc. ANGIO DYNAMICS 12401819 UNI-FUSE INFUSION CATHETER 4F X 90CM X 20CM. December 2024.

53.	DOTmed.com Inc. MERIT MEDICAL IS4-135-20 Fountain Infusion Catheter. 2024.

54.	Potter BJ and Ann Thompson C. Cost of operating room time for endovascular transcatheter aortic valve replacement. *Journal of Medical Economics*. 2019;22:1022-1024.

55.	Childers CP and Maggard-Gibbons M. Understanding costs of care in the operating room. *JAMA surgery*. 2018;153:e176233-e176233.

56.	(KFF) KFF. Hospital Adjusted Expenses per Inpatient Day by Ownership, $3167 cost per day for non-profit hospitals in US. 2022.

57.	Beckers Hospital Review. Average cost per inpatient day across 50 states.

58.	Shaffer EE, Pham A, Woldman RL, Spiegelman A, Strassels SA, Wan GJ and Zimmerman T. Estimating the effect of intravenous acetaminophen for postoperative pain management on length of stay and inpatient hospital costs. *Advances in therapy*. 2016;33:2211-2228.

59.	Ohsfeldt R, Kelton K, Klein T, Belger M, Mc Collam PL, Spiro T, Burge R and Ahuja N. Cost-effectiveness of baricitinib compared with standard of care: a modeling study in hospitalized patients with COVID-19 in the United States. *Clinical Therapeutics*. 2021;43:1877-1893. e4.

60.	Halpern NA, Goldman DA, Tan KS and Pastores SM. Trends in critical care beds and use among population groups and Medicare and Medicaid beneficiaries in the United States: 2000–2010. *Critical care medicine*. 2016;44:1490-1499.

61.	Gershengorn HB, Garland A and Gong MN. Patterns of daily costs differ for medical and surgical intensive care unit patients. *Annals of the American Thoracic Society*. 2015;12:1831-1836.

62.	Amin A, Deitelzweig S, Bucior I, Lin J, Lingohr-Smith M, Menges B and Neuman WR. Frequency of hospital readmissions for venous thromboembolism and associated hospital costs and length of stay among acute medically ill patients in the US. *Journal of Medical Economics*. 2019;22:1119-1125.

63.	Spyropoulos AC and Lin J. Direct medical costs of venous thromboembolism and subsequent hospital readmission rates: an administrative claims analysis from 30 managed care organizations. *Journal of Managed Care Pharmacy*. 2007;13:475-486.

64.	Bullano MF, Willey V, Hauch O, Wygant G, Spyropoulos AC and Hoffman L. Longitudinal evaluation of health plan cost per venous thromboembolism or bleed event in patients with a prior venous thromboembolism event during hospitalization. *Journal of Managed Care Pharmacy*. 2005;11:663-673.

65.	Jiang HJ and Hensche M. Healthcare cost and utilization project (HCUP) statistical brief #304 - Characteristics Of 30-Day All-Cause Hospital Readmissions, 2016-2020. *Agency for Healthcare Research and Quality (US)*. 2006.

66.	Kracalik I, Sapiano MR, Wild RC, Chavez Ortiz J, Stewart P, Berger JJ, Basavaraju SV and Free RJ. Supplemental findings of the 2021 National Blood Collection and Utilization Survey. *Transfusion*. 2023;63:S19-S42.

67.	Drugs.com. Eliquis (apixaban) prices, $567 for 60 tablets (5mg) in June, 2022. 2022.

68.	Drugs.com. Eliquis (apixaban) dosage by indication. 2022.

69.	Ariesen M, Claus S, Rinkel G and Algra A. Risk factors for intracerebral hemorrhage in the general population: a systematic review. *Stroke*. 2003;34:2060-2065.

70.	Rajsic S, Gothe H, Borba H, Sroczynski G, Vujicic J, Toell T and Siebert U. Economic burden of stroke: a systematic review on post-stroke care. *The European Journal of Health Economics*. 2019;20:107-134.

71.	Fairman KA, Davis LE, Kruse CR and Sclar DA. Financial impact of direct-acting oral anticoagulants in medicaid: budgetary assessment based on number needed to treat. *Applied health economics and health policy*. 2017;15:203-214.

72.	Kirson NY, Birnbaum HG, Ivanova JI, Waldman T, Joish V and Williamson T. Excess costs associated with patients with pulmonary arterial hypertension in a US privately insured population. *Applied health economics and health policy*. 2011;9:293-303.

73.	Kirson NY, Birnbaum HG, Ivanova JI, Waldman T, Joish V and Williamson T. Excess costs associated with patients with chronic thromboembolic pulmonary hypertension in a US privately insured population. *Applied health economics and health policy*. 2011;9:377-387.

74.	Said Q, Martin BC, Joish VN, Kreilick C and Mathai SC. The cost to managed care of managing pulmonary hypertension. *Journal of medical economics*. 2012;15:500-508.

75.	Jiang R, Janssen MB and Pickard AS. US population norms for the EQ-5D-5L and comparison of norms from face-to-face and online samples. *Quality of Life Research*. 2021;30:803-816.

76.	Stokes EA, Lazaroo MJ, Clout M, Brett SJ, Black S, Kirby K, Nolan JP, Reeves BC, Robinson M and Rogers CA. Cost-effectiveness of the i-gel supraglottic airway device compared to tracheal intubation during out-of-hospital cardiac arrest: Findings from the AIRWAYS-2 randomised controlled trial. *Resuscitation*. 2021;167:1-9.

77.	Elliott VJ, Rodgers DL and Brett SJ. Systematic review of quality of life and other patient-centred outcomes after cardiac arrest survival. *Resuscitation*. 2011;82:247-256.

78.	Schluep M, Hoeks SE, Blans M, van den Bogaard B, Koopman-van Gemert A, Kuijs C, Hukshorn C, van der Meer N, Knook M and van Melsen T. Long-term survival and health-related quality of life after in-hospital cardiac arrest. *Resuscitation*. 2021;167:297-306.

79.	Qazi AH, Kennedy K, Bradley SM and Chan PS. Impact of timing of cardiac arrest during hospitalization on survival outcomes and subsequent length of stay. *Resuscitation*. 2017;121:117-122.

80.	Walter E, Berger T, Bajer-Kornek B and Deisenhammer F. Cost-utility analysis of alemtuzumab in comparison with interferon beta, fingolimod, and natalizumab treatment for relapsing-remitting multiple sclerosis in Austria. *Journal of Medical Economics*. 2019;22:226-237.

81.	Soini E, Joutseno J and Sumelahti M-L. Cost-utility of first-line disease-modifying treatments for relapsing–remitting multiple sclerosis. *Clinical therapeutics*. 2017;39:537-557. e10.

82.	Morris PE, Berry MJ, Files DC, Thompson JC, Hauser J, Flores L, Dhar S, Chmelo E, Lovato J and Case LD. Standardized rehabilitation and hospital length of stay among patients with acute respiratory failure: a randomized clinical trial. *Jama*. 2016;315:2694-2702.

83.	Tafazzoli A, Reifsnider OS, Bellanca L, Ishak J, Carrasco M, Rakonczai P, Stargardter M and Linden S. A European multinational cost-effectiveness analysis of empagliflozin in heart failure with reduced ejection fraction. *The European Journal of Health Economics*. 2023;24:1441-1454.

84.	Vadia R, Malyar N and Stargardt T. Cost-utility analysis of early versus delayed endovascular intervention in critical limb-threatening ischemia patients with rest pain. *Journal of Vascular Surgery*. 2023;77:299-308. e2.

85.	King JB, Shah RU, Bress AP, Nelson RE and Bellows BK. Cost-effectiveness of sacubitril-valsartan combination therapy compared with enalapril for the treatment of heart failure with reduced ejection fraction. *JACC: Heart Failure*. 2016;4:392-402.

86.	Liang L, Bin-Chia Wu D, Aziz MIA, Wong R, Sim D, Leong KTG, Wei YQ, Tan D and Ng K. Cost-effectiveness of sacubitril/valsartan versus enalapril in patients with heart failure and reduced ejection fraction. *Journal of Medical Economics*. 2018;21:174-181.

87.	Yao G, Freemantle N, Flather M, Tharmanathan P, Coats A and Poole-Wilson PA. Long-term cost-effectiveness analysis of nebivolol compared with standard care in elderly patients with heart failure: an individual patient-based simulation model. *Pharmacoeconomics*. 2008;26:879-889.

88.	Mealing S, Feldman T, Eaton J, Singh M and Scott DA. EVEREST II high risk study based UK cost-effectiveness analysis of MitraClip® in patients with severe mitral regurgitation ineligible for conventional repair/replacement surgery. *Journal of medical economics*. 2013;16:1317-1326.

89.	Klarenbach S, Lee K, Boucher M, So H, Manns B and Tonelli M. Direct oral anticoagulants for the treatment of venous thromboembolic events: economic evaluation. 2016.

90.	Bamber L, Muston D, McLeod E, Guillermin A, Lowin J and Patel R. Cost-effectiveness analysis of treatment of venous thromboembolism with rivaroxaban compared with combined low molecular weight heparin/vitamin K antagonist. *Thrombosis journal*. 2015;13:1-12.

91.	Gourzoulidis G, Kourlaba G, Kakisis J, Matsagkas M, Giannakoulas G, Gourgoulianis KI, Vassilakopoulos T and Maniadakis N. Cost-effectiveness analysis of rivaroxaban for treatment of deep vein thrombosis and pulmonary embolism in Greece. *Clinical Drug Investigation*. 2017;37:833-844.

92.	Heisen M, Treur MJ, Heemstra HE, Giesen EB and Postma MJ. Cost-effectiveness analysis of rivaroxaban for treatment and secondary prevention of venous thromboembolism in the Netherlands. *Journal of Medical Economics*. 2017;20:813-824.

93.	Locadia M, Bossuyt PM, Stalmeier PF, Sprangers MA, van Dongen CJ, Middeldorp S, Bank I, van der Meer J, Hamulyák K and Prins MH. Treatment of venous thromboembolism with vitamin K antagonists: patients’ health state valuations and treatment preferences. *Thrombosis and haemostasis*. 2004;92:1336-1341.

94.	Hogg K, Kimpton M, Carrier M, Coyle D, Forgie M and Wells P. Estimating quality of life in acute venous thrombosis. *JAMA internal medicine*. 2013;173:1067-1072.

95.	Lamers LM. The transformation of utilities for health states worse than death: consequences for the estimation of EQ-5D value sets. *Medical care*. 2007;45:238-244.

96.	Thom HH, Hollingworth W, Sofat R, Wang Z, Fang W, Bodalia PN, Bryden PA, Davies PA, Caldwell DM and Dias S. Directly acting oral anticoagulants for the prevention of stroke in atrial fibrillation in England and Wales: cost-effectiveness model and value of information analysis. *MDM policy & practice*. 2019;4:2381468319866828.

97.	Sullivan PW, Slejko JF, Sculpher MJ and Ghushchyan V. Catalogue of EQ-5D scores for the United Kingdom. *Medical Decision Making*. 2011;31:800-804.

98.	Dorian P, Kongnakorn T, Phatak H, Rublee DA, Kuznik A, Lanitis T, Liu LZ, Iloeje U, Hernandez L and Lip GY. Cost-effectiveness of apixaban vs. current standard of care for stroke prevention in patients with atrial fibrillation. *European heart journal*. 2014;35:1897-1906.

99.	Page MJ, McKenzie JE, Bossuyt PM, Boutron I, Hoffmann TC, Mulrow CD, Shamseer L, Tetzlaff JM, Akl EA and Brennan SE. The PRISMA 2020 statement: an updated guideline for reporting systematic reviews. *bmj*. 2021;372.

100.	Johnson K, VandenHull A, Remund T, Pohlson K, Bares V, Wacker J and Kelly P. Short-term cost comparison of systemic heparin therapy vs. catheter directed thrombolysis for the treatment of massive and submassive pulmonary embolism with long-term chronic pulmonary hypertension cost model. *South Dakota medicine: the journal of the South Dakota State Medical Association*. 2021;74:70.

101.	Colombo C, Capsoni N, Russo F, Iannaccone M, Adamo M, Viola G, Bossi IE, Villanova L, Tognola C and Curci C. Ultrasound-Assisted, Catheter-Directed Thrombolysis for Acute Intermediate/High-Risk Pulmonary Embolism: Design of the Multicenter USAT IH-PE Registry and Preliminary Results. *Journal of Clinical Medicine*. 2024;13:619.

102.	Anderson J, Heidenreich P, Barnett P, Creager M, Fonarow G, Gibbons R, Halperin J, Hlatky M, Jacobs A and Mark D. AAC/AHA task force on performance measures; ACC/AHA task force on practice guidelines. ACC/AHA statement on cost/value methodology in clinical practice guidelines and performance measures: a report of the American College of Cardiology/American Heart Association task force on performance measures and task force on practice guidelines. *J Am Coll Cardiol*. 2014;63:2304-22.
